# Vaccine-associated complications: a comparative multicenter evaluation among dental practitioners and dental students—which candidate vaccine is more safe in SARS COV II, Gam-COVID-Vac (Sputnik V), ChAdOx1 nCoV-19 (AstraZeneca), BBV152 (Covaxin), or BBIBP-CorV(Sinopharm)?

**DOI:** 10.1186/s40902-021-00330-6

**Published:** 2022-01-13

**Authors:** Behzad Houshmand, Seied Omid Keyhan, Hamid Reza Fallahi, Shaqayeq Ramezanzade, Erfan Sadeghi, Parisa Yousefi

**Affiliations:** 1 School of Dentistry, Shahid Beheshti University of Medical Sciences, Tehran, Iran; 2 College of Medicine, University of Florida, Jacksonville, Florida USA; 3 Maxillofacial Surgery Implantology & Biomaterial Research Foundation Tehran, Isfahan, Iran; 4 Department of Biostatistics and Epidemiology, School of Health, Isfahan University of Medical Sciences, Isfahan, Iran; 5 Department of Prosthodontics, Dental College, Isfahan University of Medical Science, Isfahan, Iran

**Keywords:** Vaccine, SARS-COV2, Side effect

## Abstract

**Background:**

The rapidly developed vaccines against the severe acute respiratory syndrome coronavirus 2 carry a risk of provoking side effects. This study aimed to evaluate current vaccination non-serious/serious side effects.

**Methods:**

A multicenter electronic questionnaire via an online platform was conducted over a 1-week period among vaccinated dental staff and dental students inquiring whether they experienced vaccine-related side-effects after vaccine administration.

**Results:**

A total of 1205 respondents with a mean age of 39 (SD: 12) were retained for the analyses. The following vaccines were reported; Gam-COVID-Vac (Sputnik V), ChAdOx1 nCoV-19 (AstraZeneca), BBV152 (Covaxin), or BBIBP-CorV (Sinopharm). The majority of respondents received ChAdOx1 nCoV-19 (51.1%) and Gam-COVID-Vac (37.6%). The symptoms most frequently reported after vaccination were fatigue (79%), local pain in the injection site (77.4%), malaise (73%), and body pain (71.1%). Enrollees reported more onset of reactions on 0–12 h (44.1%) and 12–24 h (29.0%) after vaccine administration (*p* value <0.001). In 75.7%, the side effects last for up to 3 days. Merely 5.5% of cases reported the presence of side effects after the first week. Individuals with a history of SARSCoV-2 and other infections (MERS, influenza, and EBV) were more likely to report a number of unserious systemic side effects.

**Conclusion:**

The commonly reported adverse events were in line with similar studies. We have concerns with the frequency of serious adverse effects. This work necessitates the need for further clinical assessments with larger sample sizes.

**Supplementary Information:**

The online version contains supplementary material available at 10.1186/s40902-021-00330-6.

## Introduction

In late 2019, the severe acute respiratory syndrome coronavirus 2 (SARSCoV-2), the cause of COVID-19 disease, emerged in Wuhan, China [1]. On March 11, 2020, this new human viral pathogen reached pandemic status. In February 19, the first cases of SARSCoV-2 were officially announced in Qom city, Iran [2]. In February 11, 2021, Iran launches vaccination against SARS-Cov-2 starting with Gam-COVID-Vac (Sputnik V) administration to the frontline medical workers [3]. Iran issued permit for emergency use for Gam-COVID-Vac, ChAdOx1 nCoV-19 (AstraZeneca), BBV152 (Covaxin) candidate vaccines on February 17, 2021, and BBIBP-CorVvaccine (Sinopharm) on March 10, 2021 [4, 5]. By the end of April 2021, more than 137 million SARSCoV-2 cases and almost 3 million confirmed deaths were registered worldwide [6].

During the early phase of the SARSCoV-2 pandemic, non-pharmaceutical interventions and social distancing strategies, at the cost of reduced economic activities, were advocated to mitigate the SARSCoV-2 transmission [7]. Vaccination, instead, is a viable indirect protection to increase the immunity of healthcare workers and the general population against SARS-CoV-2 infection.

Due to frequent close face-to-face contact with patients and frequent exposure to contaminated body fluids such as respiratory tract secretions and saliva, dental healthcare workers are at increased risk for exposure to SARSCoV-2 infection [8]. Therefore, achieving high vaccine coverage rates among this group is warranted [9].

Despite the vaccines having been approved and rolled out to millions worldwide, the rapidly developed vaccines also carry a risk of provoking side effects.

By June 2021, the regionally available vaccines across Iran have been ChAdOx1 nCoV-19, Gam-COVID-Vac, BBV152, and BBIBP-CorV. The first two (ChAdOx1 nCoV-19 and Gam-COVID-Vac) are both adenoviral-based vector vaccines with a phase 3 reported efficacy of 76% (at 22–90 days) [10] and 91% efficacy at 21 days after at least one standard dose [11], respectively. While BBV152 and BBIBP-CorV are inactivated vaccines with a reported a clinical efficacy of 81% [12] and 79% [13], respectively.

The question of how safe are the SARSCoV-2 vaccines has loomed large globally. Currently, side effects of vaccines are not fully evaluated and robust data regarding what vaccine recipients might experience is lacking. More precise data on common, serious as well as unexpected side effects of available vaccines are warranted to address vaccine hesitancy and to provide reassurance [14].

To the best of the authors’ knowledge, this is the first study to provide detailed information both on the frequency and intensity of a wide range of potential side effects related to four different types of candidate vaccines authorized emergently and currently available in our region and compares side effects across vaccines. This study aimed to evaluate current vaccination non-serious/serious side effects among dental staff across the country who receive available SARS-CoV-2 vaccines in Iran.

The secondary aim was to assess any relationship between a history of previous viral infections including SARSCoV-2, influenza, Middle East Respiratory Syndrome (MERS), Epstein-Barr virus (EBV), and frequency of side effects after vaccination.

## Materials and methods

### Study design

This study followed the American Association for Public Opinion Research (AAPOR) reporting guideline [15], and the study protocol was registered at Shahid Beheshti University of Medical Sciences, with registration number IR.SBMU. RETECH. REC. 1400.164.

From April 25, 2021, to May 5, 2021, we distributed a multicenter electronic questionnaire via an online platform over a 1 week period among the healthy ASA I (American Society of Anesthesiologists Classification) vaccinated dental staff and dental students across the country who have received at least the first dose of one of the SARS-CoV-2 vaccines available in Iran, inquiring whether they experienced vaccine-related side-effects after vaccine administration.

### Data collection

We collected the following self-reported by responders

- Demographic data including gender (male or female), age, occupation (dental student, general dentist, postgraduate dental student, professional dentist)
- History of coronavirus infections
- History of previous viral infections including MERS, EBV (mononucleosis), influenza
- Type of vaccine: Gam-COVID-Vac, ChAdOx1 nCoV-19, BBV152, and BBIBP-CorV
- Data on doses of vaccine administered: single-dose, first dose, and booster (date of receiving last dose)
- The following signs and symptoms were included and patients were asked to rate each on a 0–10 numerical scale (with zero as no symptom and 10 as the worst symptom one has ever experienced): muscle soreness and myalgia, headache, fatigue, visual disorders, nausea and vomiting, fever, chills and shiver, local pain in the hand, pain in the foot, cellulitis warmth and swollen armpit glands, loss of appetite, dizziness, redness and itch, arthralgia, chest pain, cough, shortness of breath, Diplopia, diarrhea, insomnia, jaw pain, dysphagia, facial numbness, anesthesia (face/body), bradycardia, tachycardia, thrombosis and blood-clotting conditions, oral bleeding, nasal bleeding, faint, seizure, optic neuritis, and speech disorder
- The onset of vaccine side effects: 0–12 h
  12–24
  1–2 days
  3–4 days
  5–7 days
  1–2 weeks
  3–4 weeks
  No side effect
- How long do the side effects last?
  Few hours
  1 day
  1–3 days
  7 days
  14 weeks
  None

### Statistical analysis

In order to measure the validity and reliability of the questionnaire, the list of side effects was advised by five experts in the field. A pilot study with 30 cases was then conducted to iron out any problems in the design of the survey.

After data collection through a 1-week period, data were presented as mean ± standard deviation or number (percent). The normality assumption of the quantitative variables was assessed using the Kolmogorov-Smirnov test. Mann-Whitney, Kruskal-Wallis, and chi-square tests were applied to compare the study variables and Spearman’s coefficient was used to investigate the linear correlations.

We used IBM SPSS Statistics version 24 (IBM Corporation, Armonk, New York, USA) and GraphPad Prism version 8 (GraphPad Software, La Jolla, CA, USA) for all statistical analyses.

## Results

### Study characteristics

Table 1 illustrates the demographic characteristic of the study population. We included 1,205 vaccinated individuals, with a mean age of 39 years (SD=12), mainly females (60.2%).

**Table 1:** The demographic data of included cases

	Number	Percent
Gender (female)	681	60.2%
Occupation		
Dental student	104	9.2%
Postgraduate dental student	178	15.7%
General dentist	831	73.4%
Professional dentist	19	1.7%
History of coronavirus infections	269	23.8%
Infected once	245	21.6%
Infected more than once	24	2.2%
History of previous viral infections		
Influenza	318	28.1%
MERS,	1	0.1%
EBV (mononucleosis)	17	1.5%
None	796	70.3%
Type of administered vaccine		
ChAdOx1 nCoV-19	578	51.1%
Gam-COVID-Vac	426	37.6%
BBV152	25	2.2%
BBIBP-CorV	102	9.0%
Onset of vaccine side effects:		
0–12 h	499	44.1%
12–24 h	328	29.0%
1–2 d	96	8.5%
3–4 d	19	1.7%
5–7 d	9	0.8%
1–2 w	12	1.1%
3–4 w	3	0.3%
No side effect	166	14.7%
How long after vaccine that you have side effects (duration of side effects)		
Few hours	191	16.9%
1 d	498	44.0%
1–3 d	358	31.6%
3–7 d	48	4.2%
7–14 d	10	0.9%
>14 d	4	0.4%
None	23	2.0%

Abbreviations: *h* hour, *d* day, *w* week

The majority of respondents reported receiving a single dose of vaccine (93.0%). ChAdOx1 nCoV-19 (51.1%) and Gam-COVID-Vac (37.6%) were mostly administered. 44.1% of cases reported an onset of 0–12 h after vaccination, 29.0% during the 12–24 h after vaccination while 14.7% of cases reported no side effects.

### Frequency and intensity of side effects

Available information from our analysis suggested a statistically highly significant relationship (*P* value <0.001) between SARSCoV-2 vaccination and the following symptoms (Additional file 1): fatigue, malaise, headache, body pain, vision disorder, fever, chills and shiver, local pain in the hand, pain in the foot, loss of appetite, dizziness, crump/abdominal pain, arthralgia, chest pain, cough, shortness of breath, blurred vision, diarrhea, insomnia, jaw pain, earache, and bradycardia. And a statistically significant relationship (*P* value <0.05) between SARSCoV-2 vaccination and the following symptoms (Additional file 1):

### Vomiting, redness and itch, and Diplopia

**Fig. 1:**
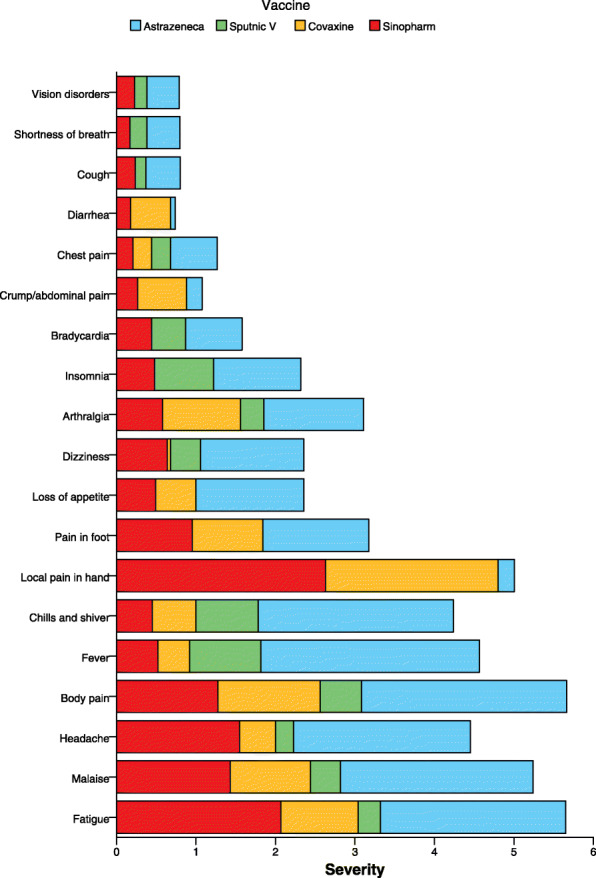
The symptoms’ intensity on 0–10 Numeric Rating Scale in four types of vaccine; ChAdOx1 nCoV-19, Gam-COVID-Vac, BBV152, and BBIBP-CorV

**Fig. 2:**
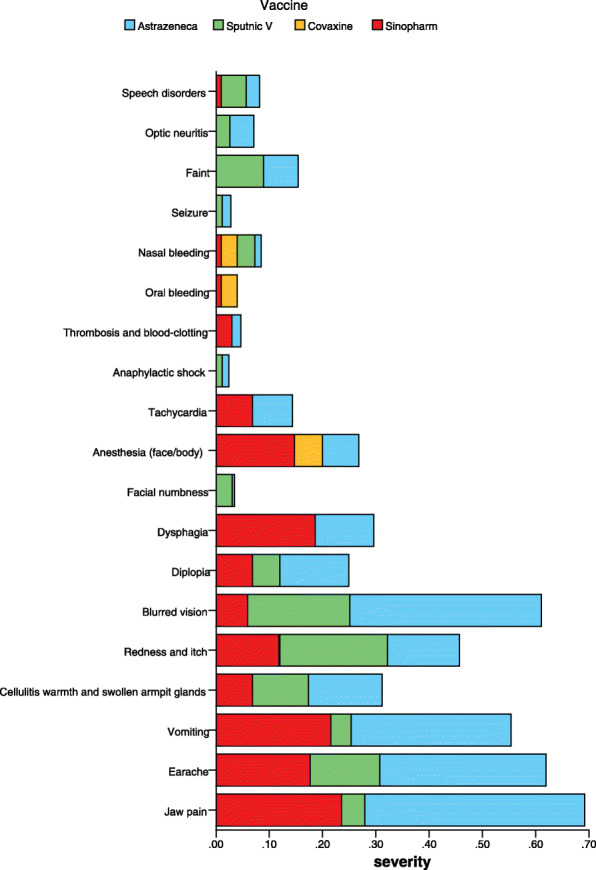
Continue of the symptoms’ intensity on 0–10 Numeric Rating Scale in four types of vaccine; ChAdOx1 nCoV-19, Gam-COVID-Vac, BBV152, and BBIBP-CorV

The symptoms most frequently reported after vaccination were fatigue (79%), local pain in the injection site (77.4%), malaise (73%), and body pain (71.1%) (Figs. 1 and 2 and Table 2).

**Table 2:** Frequency of side effects in total

		Number	Percent (%)
Fatigue	No	238	21.0%
	Yes	894	79.0%
Malaise	No	306	27.0%
	Yes	826	73.0%
Headache	No	445	39.3%
	Yes	687	60.7%
Body pain	No	327	28.9%
	Yes	805	71.1%
Fever	No	492	43.5%
	Yes	640	56.5%
Chills and shiver	No	585	51.7%
	Yes	547	48.3%
Local pain in hand	No	256	22.6%
	Yes	876	77.4%
Pain in foot	No	650	57.4%
	Yes	482	42.6%
Loss of appetite	No	716	63.3%
	Yes	416	36.7%
Dizziness	No	691	61.0%
	Yes	441	39.0%
Arthralgia	No	625	55.2%
	Yes	507	44.8%
Insomnia	No	728	64.3%
	Yes	404	35.7%
Bradycardia	No	811	71.6%
	Yes	321	28.4%
Crump/abdominal pain	No	903	79.8%
	Yes	229	20.2%
Chest pain	No	875	77.3%
	Yes	257	22.7%
Diarrhea	No	971	85.8%
	Yes	161	14.2%
Cough	No	930	82.2%
	Yes	202	17.8%
Shortness of breath	No	936	82.7%
	Yes	196	17.3%
Vision disorders	No	934	82.5%
	Yes	198	17.5%
Jaw pain	No	992	87.6%
	Yes	140	12.4%
Earache	No	1003	88.6%
	Yes	129	11.4%
Vomiting	No	1018	89.9%
	Yes	114	10.1%
Cellulitis warmth and swollen armpit glands	No	1034	91.3%
	Yes	98	8.7%
Redness and itch	No	1000	88.3%
	Yes	132	11.7%
Blurred vision	No	993	87.7%
	Yes	139	12.3%
Diplopia	No	1062	93.8%
	Yes	70	6.2%
Dysphagia	No	1060	93.6%
	Yes	72	6.4%
Facial numbness	No	1112	98.2%
	Yes	20	1.8%
Anesthesia (face/body)	No	1056	93.3%
	Yes	76	6.7%
Tachycardia	No	1089	96.2%
	Yes	43	3.8%
Anaphylactic shock	No	1121	99.0%
	Yes	11	1.0%
Thrombosis and blood-clotting	No	1110	98.1%
	Yes	22	1.9%
Oral bleeding	No	1116	98.6%
	Yes	16	1.4%
Nasal bleeding	No	1095	96.7%
	Yes	37	3.3%
Seizure	No	1122	99.1%
	Yes	10	0.9%
Faint	No	1100	97.2%
	Yes	32	2.8%
Optic neuritis	No	1104	97.5%
	Yes	28	2.5%
Speech disorders	No	1098	97.0%
	Yes	34	3.0%

Figures 1 and 2 illustrate the symptoms’ intensity on 0–10 Numeric Rating Scale in four types of vaccine; ChAdOx1 nCoV-19, Gam-COVID-Vac, BBV152, and BBIBP-CorV. In total, the severity of side effects was higher in the ChAdOx1 nCoV-19 group compared with other groups (Gam-COVID-Vac, BBV152, and BBIBP-CorV). (For more information please check Additional file 1).

In our study, rare cases of serious events after vaccination were reported; 20 cases of thrombosis and blood clotting (10 ChAdOx1 nCoV-19, 7 Gam-COVID-Vac, and 3 BBIBP-CorV groups), one moderate, and one serious (both were reported in ChAdOx1 nCoV-19 group). Also 10 cases of mild allergic reactions (6 in ChAdOx1 nCoV-19 and 4 in Gam-COVID-Vac group) and 1 moderate level (ChAdOx1 nCoV-19) were reported.

### Onset and duration of side effects

Enrollees reported more onset of reactions on 0–12 h (44.1%) and 12–24 h (29.0%) after vaccine administration (*p* value <0.001). (Additional file 1)

The side effects were temporary in most cases; in 498 cases (44.0%), the side effects last for 1 day. 31.6% of cases experienced the symptoms for 1–3 days. Merely 5.5% of cases reported the presence of side effects after the first week (Additional file 1).

We considered the cases in two age groups; ≤40 and >40 years. In both groups, data regarding the onset and the duration of side effects were statistically significant with 0–12 h and 1–3 days being the most reported times, respectively.

The data for the duration of side effects based on gender is shown in Additional file 1. In both genders, the durations of side effects were statistically significant. In both groups, data regarding the onset and the duration of side effects were statistically significant with 0–12 h and 1–3 days being the most reported times, respectively.

### History of SARSCoV-2 infection

269 of 1205 cases (23.8%) reported a history of SARS-CoV-2 infection. The logistic regression model using backward method adjust for confounders (age and gender) demonstrated that individuals with a history of SARS-CoV-2 infection in ChAdOx1 nCoV-19 group were more likely to report loss of appetite, cellulitis warmth and swollen armpit glands, chest pain compared with those without known past SARS-CoV-2 infection OR (odds ratio) with 95%CI (confidence interval): 1.616 (1.052, 2.483), 1.810 (1.004, 3.265), 1.738 (1.082, 2.794), respectively.

Individuals with a history of SARS-CoV-2 infection in Gam-COVID-Vac group were more likely to report chills and shivers, and faint compared with those without known past SARS-CoV-2 infection OR with 95%CI 2.159 (1.223, 3.809), and 4.530 (1.192, 17.209), respectively.

Cases with previous SARS-CoV-2 infection in the BBIBP-CorV group reported more body pain than cases without a known history of SARS-CoV-2 infection OR (95%CI) 3.121 (1.105, 8.819). Table 3 illustrates the data regarding patients with/without a history of SARSCoV-2 infection.

**Table 3:** The data regarding patients with/without a history of SARSCoV-2 infection

Side effect	Total ***N***=	ChAdOx1 nCoV-19 ***N***=	Gam-COVID-Vac ***N***=	BBIBP-CorV ***N***=
**Facial numbness**	0.155 (0.020, 1.172)			
**Loss of appetite(1)**	1.290 (0.944, 1.763)	1.616 (1.052, 2.483)		
**Cellulitis warmth and swollen armpit glands**	1.565 (0.990, 2.472)	1.810 (1.004, 3.265)		
**Fatigue**		0.431 (0.222, 0.836)		
**Chest pain**		1.738 (1.082, 2.794)		
**Shortness of breath**		0.549 (0.323, 0.935)	1.929 (0.893, 4.166)	
**Chills and shiver**	1.298 (0.945, 1.784)		2.159 (1.223, 3.809)	
**Local pain in the hand**			1.700 (0.941, 3.072)	
**Insomnia**			1.701 (0.965, 2.998)	
**Vomiting**			0.336 (0.106, 1.063)	
**Dysphagia**			0.194 (0.045, 0.845)	
**Tachycardia**			0.187 (0.032, 1.084)	
**Faint**			4.530 (1.192, 17.209)	
**Headache**				0.350 (0.119 , 1.031)
**Body pain**				3.121 (1.105 , 8.819)

OR (95% CI), analysis adjusted for age and gender

### History of other viral infections (MERS, influenza, and EBV)

336 cases (27.8%) reported a history of MERS, influenza, and EBV before vaccine administration. In total, patients who reported a history of previous viral infections (MERS, influenza, and EBV) had significantly higher rates for cellulitis warmth and swollen armpit glands and faint (Table 4) (*P* value <0.001) and for the following side effects (*P* value<0.05): headache, chest pain, cough, and shortness of breath

**Table 4:** The relationship between a history of MERS, influenza, or EBV and the intensity of side effects (on a 0–10 scale)

Side effect	History of MERS, influenza, or EBV (mean ±standard deviation)		***P*** value
	No	Yes	
Cellulitis warmth and swollen armpit glands	0±1	0±1	<0.001
Faint	0±0	0±0	0.001
Chest pain	1±2	1±2	0.013
Shortness of breath	1±2	1±2	0.015
Cough	1±1	1±2	0.036
Headache	3±4	4±4	0.041
Loss of appetite	2±3	2±3	0.579
Anaphylactic shock	0±1	0±0	0.069
Bradycardia	0±1	0±1	0.079
Malaise	4±3	4±3	0.079
Chills and shiver	3±4	3±4	0.083
Tachycardia	1±2	1±2	0.086
Redness and itch	0±1	0±1	0.111
Fatigue	4±3	5±3	0.123
Facial numbness	0±1	0±1	0.128
Earache	0±2	1±2	0.129
Jaw pain	2±3	2±3	0.135
Nasal bleeding	0±0	0±0	0.136
Vomiting	0±2	0±2	0.136
Dizziness	2±3	2±3	0.152
Pain in foot	2±3	3±3	0.168
Local pain in the hand	4±3	4±3	0.198
Arthralgia	2±3	2±3	0.198
Fever	3±3	3±4	0.223
Dysphagia	0±2	0±2	0.249
Insomnia	4±4	4±4	0.303
Crump/abdominal pain	1±2	1±2	0.307
Blurred vision	0±1	0±1	0.347
Diarrhea	1±2	1±2	0.375
Thrombosis and blood-clotting	0±0	0±0	0.478
Optic neuritis	0±1	0±1	0.484
Oral bleeding	0±0	0±1	0.490
Seizure	0±0	0±1	0.502
Vision disorder	1±2	1±2	0.638
Speech disorders	0±0	0±0	0.722
Body pain	4±4	4±4	0.758
Anesthesia (face/body)	0±0	0±1	0.927
Diplopia	0±1	0±1	0.946

Available data did not suggest any causal relationship between a history of influenza, EBV, or MERS and the onset/duration of side effects (*P* value> 0.05) (table in Additional file 1).

Also, our data suggested that, regardless of the vaccine administration, individuals with a history of other viral infections (MERS, influenza, and EBV) had higher rates for a history of SARSCoV-2 infection (27.7%) compared with individuals without such history (22.1%) (*P* value 0.044).

## Discussion

The safety concern of candidate vaccines has loomed large over the past months. Providing robust data regarding possible side effects after SARSCoV-2 vaccine administration is crucial to provoke trust and confidence in any type of vaccine [14]. In this multicenter questionnaire-based survey among dental students and dental practitioners, we have investigated adverse effects following the administration of the four available SARSCoV-2 vaccines in Iran; ChAdOx1 nCoV-19, Gam-COVID-Vac, BBV152, and BBIBP-CorV and report that the most common reactions, in our community analysis, were fatigue, local pain in the hand, and malaise which affected 79.0%, 77.4%, and 73.0% of cases, respectively.

Individuals vaccinated with the ChAdOx1 nCoV-19 vaccine (AZD1222) were more likely to experience the aforementioned side effects compared with other groups; the mean±standard deviations were 6±3, 6±4, 5±3, and 5±3, respectively.

The most common adverse events reported for both doses of Gam-COVID-Vac were in line with another trial on health care workers [16] (pain at the injection site, fever, and muscle pain).

The incidence of events attributed to the Gam-COVID-Vac was 64.7% in phase 3 clinical trial [10].

Comparing with phase 3 clinical trial of the Gam-COVID-Vac, which reported 94% of adverse events (a total of 7966 events) as mild (10), our study population experienced higher intensity for unserious side effects (Figs. 1 and 2).

93.2% of vaccinated population experienced at least one side effect. 7 cases of thrombosis and blood clotting (1.64%) and 4 mild allergic reactions (0.93%) were reported after Gam-COVID-Vac administration.

In the phase 3 clinical trial, 0.3% of the vaccinated group had serious adverse events, although no serious side effect was considered related to the vaccine administration [10]. There was a significant discrepancy between the thrombosis rate reported in phase 3 (three cases of renal colic and deep vein thrombosis associated with pre-existing comorbidities in 21,977 included cases) and our findings (7 cases of thrombosis in 426 cases). Phase 3 suggested no association between serious adverse events and vaccine administration, but our results do not support this and further clinical assessments are highly recommended.

The ChAdOx1 nCoV-19 vaccine (AZD1222) was administered by 578 of 1205 cases of which 100.0% received only the first injection. 98.6% of ChAdOx1 nCoV-19 vaccinated population experienced at least one side effect.

10 cases of mild and one case of moderate thrombosis and blood clotting were reported. Also, 6 cases reported mild and one reported moderate levels of allergic reaction. In phase 3 clinical trial of this candidate vaccine [17] 88% of participants, aged 18–55, administered prime vaccine reported incidence of systemic adverse effects. Meanwhile, a prospective observational study in the UK reported a significantly lower rate of adverse effects (33.7%) [18].

In our study, the data for BBV152, also known as COVAXIN®, as a purified inactivated whole virion was scarce. 100% of the vaccinated population experienced at least one side effect. 16 out of 25 cases received two doses of BBV152. Individuals who received BBV152 were less likely to experience moderate levels of side effects compared with Gam-COVID-Vac and ChAdOx1 nCoV-19 group (Figs. 1 and 2). Upon cell entry, the adenovirus-vector vaccines (Gam-COVID-Vac and ChAdOx1 nCoV-19) risen the release of cytokines and chemokines causing higher levels of side reactions after vaccination compared with inactivated vaccines (BBIBP-CorV and BBV152) [19].

Among the reported side effects, the most intense and most common side effect was local pain in the hand. The interim results of phase 2 of BBV152 reporting pain at the injection site as the most common adverse event (11 of 380 patients) followed by headache, fatigue, and fever [20] although the data for phase 3 trial have not yet been published/available. Same as phase 2, in our study, no serious event was reported after BBV152 vaccination. Our initial experience is similar to currently limited literature suggesting that BBV152 is a safe and tolerable candidate vaccine with minimal and minor adverse events profile [20, 21].

The Chinese inactivated vaccine candidate, BBIBP-CoV (Beijing,China), was administered to merely 9% of our study population. 88.2% received a single dose of BBIBP-CoV. 87.3% of the vaccinated population experienced at least one side effect. The phase 2 trials reported side effects were mild in severity with no serious adverse event (21) [22]. In our study, 3 cases of thrombosis and blood clotting were reported. The current literature regarding the BBV152 is not powered to address safety and adverse events; therefore, we were unable to draw a solid conclusion.

We have concerns with the number of serious adverse effects reported: 20 cases of thrombosis and blood clotting, 12 ChAdOx1 nCoV-19, 7 Gam-COVID-Vac, and 3 BBIBP-CorV groups, were reported. The Phase 3 of Gam-COVID-Vac suggested no association between serious adverse events and vaccine administration (10). Likewise, phase 3 trials of BBIBP-CorV have not yet been published/available [21].

In April 2021, 86 potential cases of thrombosis and clots, out of 25 million vaccinated people, were reported. The blood clots have been tentatively linked to a syndrome causing unwanted immune response against platelet factor 4 after administration of adenoviral vector vaccines. There is a possibility that phase 3 reports of clotting are susceptible to biases and higher numbers of reports are expected in the near future [23]. Despite the fact that the findings of a questionnaire-based survey is not powered to address serious side effects after vaccination, it necessitates the need for further clinical assessments with large sample sizes.

The duration of side effects based on the type of administered vaccine is as below:

Most cases in groups of ChAdOx1 nCoV-19, Gam-COVID-Vac, and BBV152 experienced adverse events for a duration of 1–3 days, 66.3%, 49%, and 57%, respectively, while 53.8% of BBIBP-CorV group experienced side effects for only a few hours.

Also, the onsets of side effects were mostly 0–12 h after vaccine administration for both genders and the durations of side effects were mostly 1–3 days for both males and females.

Individuals expressing a history of SARS-CoV-2 infection, in ChAdOx1 nCoV-19, Gam-COVID-Vac, and BBIBP-CorV groups experienced some non-serious side effects more intense than those without known past SARS-CoV-2 infection. This finding is similar to the results of an observational study with more than 600,000 cases reporting a correlation between the history of SARS-CoV-2 infection and the systemic side effects.

The results from small preprints suggest that higher reactogenicity and clear antibody response, peaked almost 7 to 14 days after vaccine administration in individuals with pre-existing immunity causes higher frequencies of systemic side effects such as chills, fever, fatigue, headache, and muscle or joint pains compared to cases exposed to SARS-CoV-2 spike protein for the very first time [24, 25].

1.5% of cases (17 cases) reported a history of EBV (mononucleosis) infection. Some studies suggested the possibility of EBV reactivation in SARS-CoV-2 patients which might alter the clinical characteristics of SARS-CoV-2 infection and cause over-activation of the cellular immune system [26]. In our study, though, no significant relation between EBV previous infection and the levels of side effects was found. Larger sample sizes are warranted to assess the relationship.

336 cases (27.8%) reported a history of MERS or influenza or EBV before vaccine administration. In total, patients who reported history of previous viral infections (MERS, Influenza, or EBV) had significantly higher rates for cellulitis warmth and swollen armpit glands and faint (*P* value <0.001) and for the following side effects (*P* value<0.05):

### Headache, chest pain, cough, and shortness of breath

Reports have suggested a possibility that EBV reactivation might occur in SARSCoV-2 patients which might alter the clinical characteristics and cellular immune response leading to increased inflammation and fever [26]. Due to the limited number of reports in current study, any association between a history of EBV/MERS infection and the side effects of candidate vaccines remained unknown. Further studies with larger sample sizes are therefore warranted.

The results of this survey should be interpreted in the context of shortcomings. Due to the limited number of cases receiving multiple doses of candidate vaccines (7.0%), we were unable to compare the probability and intensity of side effects between the first and second doses of each group.

The number of cases reporting a previous viral infection, history of SARSCoV-2 or infections other than SARSCoV-2, was limited. Nevertheless, the history of SARSCoV-2 infection was considered based on symptoms and the capture of data on SARS-CoV-2 RT-PCR/lateral flow test results was not available in this survey.

Due to the limited number of cases receiving the second dose of each vaccine, the timing was not considered in the analyses. Even the most efficient questionnaire is unlikely to capture all the thromboembolic events. Therefore, the true incidence rate of thromboembolic events might be unknown.

This survey conducted a comparison between vector-based and inactivated candidate vaccines, although a comparison with common mRNA candidates was not available.

## Conclusion

In this multicenter questionnaire-based survey among dental students and dental practitioners, we have investigated adverse effects following the administration of the four available SARSCoV-2 vaccines in Iran; ChAdOx1 nCoV-19, Gam-COVID-Vac, BBV152, and BBIBP-CorV and report that the most common reactions in our community analysis were fatigue, local pain in the hand, and malaise which affected 79.0%, 77.4%, and 73.0% of cases, respectively. The most common adverse events reported were in line with similar studies. We have concerns with the serious adverse effects reported. The sample size is not powered to draw a viable conclusion, and therefore, further clinical assessments with larger sample sizes are warranted.

## Supplementary Information

**Additional file 1.**

## Data Availability

Additional data are available in the Additional file 1.

## Abbreviations

SARSCoV-2: The severe acute respiratory syndrome coronavirus 2
AAPOR: American Association for Public Opinion Research
ASA: American Society of Anesthesiologists
OR: Odds ratio
CI: Confidence interval
MERS: Middle East Respiratory Syndrome
EBV: Epstein-Barr virus
