# Supplementary material for: Vaccine-associated complications: a comparative multicenter evaluation among dental practitioners and dental students—which candidate vaccine is more safe in SARS COV II, Gam-COVID-Vac (Sputnik V), ChAdOx1 nCoV-19 (AstraZeneca), BBV152 (Covaxin), or BBIBP-CorV(Sinopharm)?
Source: Maxillofac Plast Reconstr Surg. 2022 Jan 13;44(1):3. doi: 10.1186/s40902-021-00330-6 (PMC8755981; doi:10.1186/s40902-021-00330-6)
Supplement: Supplementary file 1 — Additional file 1. [file 40902_2021_330_MOESM1_ESM.docx]

Figure. The symptom frequency and intensity on 0–10 Numeric Rating Scales for Symptoms in four types of vaccines.

|  | No side effect | Mild | Moderate | Severe |
| --- | --- | --- | --- | --- |
| Fatigue | 238 | 252 | 264 | 378 |
|  | 21.0% | 22.3% | 23.3% | 33.4% |
| Malaise | 306 | 250 | 255 | 321 |
|  | 27.0% | 22.1% | 22.5% | 28.4% |
| Headache | 445 | 229 | 169 | 289 |
|  | 39.3% | 20.2% | 14.9% | 25.5% |
| Body pain | 327 | 225 | 191 | 389 |
|  | 28.9% | 19.9% | 16.9% | 34.4% |
| Fever | 492 | 208 | 166 | 266 |
|  | 43.5% | 18.4% | 14.7% | 23.5% |
| Chills and shiver | 585 | 146 | 136 | 265 |
|  | 51.7% | 12.9% | 12.0% | 23.4% |
| Local pain in hand | 256 | 298 | 225 | 353 |
|  | 22.6% | 26.3% | 19.9% | 31.2% |
| Pain in foot | 650 | 141 | 145 | 196 |
|  | 57.4% | 12.5% | 12.8% | 17.3% |
| Loss of appetite | 716 | 192 | 127 | 97 |
|  | 63.3% | 17.0% | 11.2% | 8.6% |
| Dizziness | 691 | 209 | 129 | 103 |
|  | 61.0% | 18.5% | 11.4% | 9.1% |
| Arthralgia | 625 | 163 | 156 | 188 |
|  | 55.2% | 14.4% | 13.8% | 16.6% |
| Insomnia | 728 | 168 | 121 | 115 |
|  | 64.3% | 14.8% | 10.7% | 10.2% |
| Bradycardia | 811 | 162 | 87 | 72 |
|  | 71.6% | 14.3% | 7.7% | 6.4% |
| Crump/abdominal pain | 903 | 126 | 69 | 34 |
|  | 79.8% | 11.1% | 6.1% | 3.0% |
| Chest pain | 875 | 126 | 77 | 54 |
|  | 77.3% | 11.1% | 6.8% | 4.8% |
| Diarrhea | 971 | 93 | 37 | 31 |
|  | 85.8% | 8.2% | 3.3% | 2.7% |
| Cough | 930 | 125 | 56 | 21 |
|  | 82.2% | 11.0% | 4.9% | 1.9% |
| Shortness of breath | 936 | 124 | 48 | 24 |
|  | 82.7% | 11.0% | 4.2% | 2.1% |
| Vision disorders | 934 | 129 | 43 | 26 |
|  | 82.5% | 11.4% | 3.8% | 2.3% |
| Jaw pain | 992 | 74 | 35 | 31 |
|  | 87.6% | 6.5% | 3.1% | 2.7% |
| Earache | 1003 | 70 | 30 | 29 |
|  | 88.6% | 6.2% | 2.7% | 2.6% |
| Vomiting | 1018 | 67 | 21 | 26 |
|  | 89.9% | 5.9% | 1.9% | 2.3% |
| Cellulitis warmth and swollen armpit glands | 1034 | 74 | 18 | 6 |
|  | 91.3% | 6.5% | 1.6% | 0.5% |
| Redness and itch | 1000 | 87 | 34 | 11 |
|  | 88.3% | 7.7% | 3.0% | 1.0% |
| Blurred vision | 993 | 85 | 35 | 19 |
|  | 87.7% | 7.5% | 3.1% | 1.7% |
| Diplopia | 1062 | 50 | 15 | 5 |
|  | 93.8% | 4.4% | 1.3% | 0.4% |
| Dysphagia | 1060 | 44 | 20 | 8 |
|  | 93.6% | 3.9% | 1.8% | 0.7% |
| Facial numbness | 1112 | 19 | 0 | 1 |
|  | 98.2% | 1.7% | 0.0% | 0.1% |
| Anesthesia (face/body) | 1056 | 48 | 17 | 11 |
|  | 93.3% | 4.2% | 1.5% | 1.0% |
| Tachycardia | 1089 | 31 | 8 | 4 |
|  | 96.2% | 2.7% | 0.7% | 0.4% |
| Anaphylactic shock | 1121 | 10 | 1 | 0 |
|  | 99.0% | 0.9% | 0.1% | 0.0% |
| Thrombosis and blood-clotting | 1110 | 20 | 1 | 1 |
|  | 98.1% | 1.8% | 0.1% | 0.1% |
| Oral bleeding | 1116 | 14 | 2 | 0 |
|  | 98.6% | 1.2% | 0.2% | 0.0% |
| Nasal bleeding | 1095 | 31 | 3 | 3 |
|  | 96.7% | 2.7% | 0.3% | 0.3% |
| Seizure | 1122 | 9 | 0 | 1 |
|  | 99.1% | 0.8% | 0.0% | 0.1% |
| Faint | 1100 | 17 | 9 | 6 |
|  | 97.2% | 1.5% | 0.8% | 0.5% |
| Optic neuritis | 1104 | 24 | 3 | 1 |
|  | 97.5% | 2.1% | 0.3% | 0.1% |
| Speech disorders | 1098 | 29 | 3 | 2 |
|  | 97.0% | 2.6% | 0.3% | 0.2% |

Table. Table. The severity reported (percentage) for each side effect.

|  | | Vaccine | | | | | | | |
| --- | --- | --- | --- | --- | --- | --- | --- | --- | --- |
|  |  | Astrazeneca | | Sputnic V | | Covaxine | | Sinopharm | |
| Fatigue | No side effect | 53 | 9.2% | 132 | 31.0% | 7 | 28.0% | 45 | 44.1% |
|  | Mild | 101 | 17.5% | 110 | 25.8% | 8 | 32.0% | 33 | 32.4% |
|  | Moderate | 155 | 26.8% | 91 | 21.4% | 4 | 16.0% | 14 | 13.7% |
|  | Severe | 269 | 46.5% | 93 | 21.8% | 6 | 24.0% | 10 | 9.8% |
| Malaise | No side effect | 81 | 14.0% | 160 | 37.6% | 7 | 28.0% | 57 | 55.9% |
|  | Mild | 99 | 17.1% | 111 | 26.1% | 11 | 44.0% | 29 | 28.4% |
|  | Moderate | 152 | 26.3% | 90 | 21.1% | 3 | 12.0% | 10 | 9.8% |
|  | Severe | 246 | 42.6% | 65 | 15.3% | 4 | 16.0% | 6 | 5.9% |
| Headache | No side effect | 142 | 24.6% | 230 | 54.0% | 13 | 52.0% | 59 | 57.8% |
|  | Mild | 118 | 20.4% | 79 | 18.5% | 5 | 20.0% | 27 | 26.5% |
|  | Moderate | 106 | 18.3% | 53 | 12.4% | 5 | 20.0% | 5 | 4.9% |
|  | Severe | 212 | 36.7% | 64 | 15.0% | 2 | 8.0% | 11 | 10.8% |
| Body pain | No side effect | 85 | 14.7% | 169 | 39.7% | 11 | 44.0% | 61 | 59.8% |
|  | Mild | 92 | 15.9% | 98 | 23.0% | 8 | 32.0% | 27 | 26.5% |
|  | Moderate | 118 | 20.4% | 64 | 15.0% | 0 | 0.0% | 9 | 8.8% |
|  | Severe | 283 | 49.0% | 95 | 22.3% | 6 | 24.0% | 5 | 4.9% |
| Fever | No side effect | 149 | 25.8% | 249 | 58.5% | 19 | 76.0% | 74 | 72.5% |
|  | Mild | 96 | 16.6% | 83 | 19.5% | 4 | 16.0% | 25 | 24.5% |
|  | Moderate | 116 | 20.1% | 46 | 10.8% | 1 | 4.0% | 3 | 2.9% |
|  | Severe | 217 | 37.5% | 48 | 11.3% | 1 | 4.0% | 0 | 0.0% |
| Chills and shiver | No side effect | 193 | 33.4% | 284 | 66.7% | 21 | 84.0% | 86 | 84.3% |
|  | Mild | 83 | 14.4% | 52 | 12.2% | 1 | 4.0% | 10 | 9.8% |
|  | Moderate | 95 | 16.4% | 35 | 8.2% | 1 | 4.0% | 5 | 4.9% |
|  | Severe | 207 | 35.8% | 55 | 12.9% | 2 | 8.0% | 1 | 1.0% |
| Local pain in hand | No side effect | 79 | 13.7% | 139 | 32.6% | 2 | 8.0% | 35 | 34.3% |
|  | Mild | 139 | 24.0% | 115 | 27.0% | 9 | 36.0% | 35 | 34.3% |
|  | Moderate | 136 | 23.5% | 68 | 16.0% | 6 | 24.0% | 15 | 14.7% |
|  | Severe | 224 | 38.8% | 104 | 24.4% | 8 | 32.0% | 17 | 16.7% |
| Pain in foot | No side effect | 261 | 45.2% | 294 | 69.0% | 18 | 72.0% | 76 | 74.5% |
|  | Mild | 84 | 14.5% | 42 | 9.9% | 1 | 4.0% | 14 | 13.7% |
|  | Moderate | 94 | 16.3% | 42 | 9.9% | 2 | 8.0% | 7 | 6.9% |
|  | Severe | 139 | 24.0% | 48 | 11.3% | 4 | 16.0% | 5 | 4.9% |
| Loss of appetite | No side effect | 291 | 50.3% | 323 | 75.8% | 17 | 68.0% | 84 | 82.4% |
|  | Mild | 110 | 19.0% | 64 | 15.0% | 6 | 24.0% | 12 | 11.8% |
|  | Moderate | 94 | 16.3% | 26 | 6.1% | 1 | 4.0% | 6 | 5.9% |
|  | Severe | 83 | 14.4% | 13 | 3.1% | 1 | 4.0% | 0 | 0.0% |
| Dizziness | No side effect | 293 | 50.7% | 300 | 70.4% | 19 | 76.0% | 78 | 76.5% |
|  | Mild | 111 | 19.2% | 76 | 17.8% | 5 | 20.0% | 17 | 16.7% |
|  | Moderate | 90 | 15.6% | 32 | 7.5% | 1 | 4.0% | 6 | 5.9% |
|  | Severe | 84 | 14.5% | 18 | 4.2% | 0 | 0.0% | 1 | 1.0% |
| Arthralgia | No side effect | 257 | 44.5% | 270 | 63.4% | 16 | 64.0% | 81 | 79.4% |
|  | Mild | 90 | 15.6% | 55 | 12.9% | 3 | 12.0% | 15 | 14.7% |
|  | Moderate | 99 | 17.1% | 48 | 11.3% | 5 | 20.0% | 4 | 3.9% |
|  | Severe | 132 | 22.8% | 53 | 12.4% | 1 | 4.0% | 2 | 2.0% |
| Insomnia | No side effect | 314 | 54.3% | 305 | 71.6% | 23 | 92.0% | 85 | 83.3% |
|  | Mild | 97 | 16.8% | 58 | 13.6% | 1 | 4.0% | 12 | 11.8% |
|  | Moderate | 81 | 14.0% | 34 | 8.0% | 1 | 4.0% | 5 | 4.9% |
|  | Severe | 86 | 14.9% | 29 | 6.8% | 0 | 0.0% | 0 | 0.0% |
| VAR00001 | No side effect | 0 | 0.0% | 0 | 0.0% | 0 | 0.0% | 0 | 0.0% |
|  | Mild | 0 | 0.0% | 0 | 0.0% | 0 | 0.0% | 0 | 0.0% |
|  | Moderate | 0 | 0.0% | 0 | 0.0% | 0 | 0.0% | 0 | 0.0% |
|  | Severe | 0 | 0.0% | 0 | 0.0% | 0 | 0.0% | 0 | 0.0% |
| Bradycardia | No side effect | 373 | 64.5% | 332 | 77.9% | 21 | 84.0% | 84 | 82.4% |
|  | Mild | 93 | 16.1% | 50 | 11.7% | 4 | 16.0% | 15 | 14.7% |
|  | Moderate | 61 | 10.6% | 24 | 5.6% | 0 | 0.0% | 2 | 2.0% |
|  | Severe | 51 | 8.8% | 20 | 4.7% | 0 | 0.0% | 1 | 1.0% |
| Crump/abdominal pain | No side effect | 424 | 73.4% | 367 | 86.2% | 20 | 80.0% | 91 | 89.2% |
|  | Mild | 79 | 13.7% | 36 | 8.5% | 2 | 8.0% | 9 | 8.8% |
|  | Moderate | 51 | 8.8% | 15 | 3.5% | 2 | 8.0% | 1 | 1.0% |
|  | Severe | 24 | 4.2% | 8 | 1.9% | 1 | 4.0% | 1 | 1.0% |
| Chest pain | No side effect | 407 | 70.4% | 354 | 83.1% | 21 | 84.0% | 92 | 90.2% |
|  | Mild | 80 | 13.8% | 35 | 8.2% | 3 | 12.0% | 8 | 7.8% |
|  | Moderate | 53 | 9.2% | 21 | 4.9% | 1 | 4.0% | 2 | 2.0% |
|  | Severe | 38 | 6.6% | 16 | 3.8% | 0 | 0.0% | 0 | 0.0% |
| Diarrhea | No side effect | 472 | 81.7% | 378 | 88.7% | 23 | 92.0% | 97 | 95.1% |
|  | Mild | 56 | 9.7% | 34 | 8.0% | 0 | 0.0% | 3 | 2.9% |
|  | Moderate | 26 | 4.5% | 10 | 2.3% | 0 | 0.0% | 1 | 1.0% |
|  | Severe | 24 | 4.2% | 4 | 0.9% | 2 | 8.0% | 1 | 1.0% |
| Cough | No side effect | 448 | 77.5% | 367 | 86.2% | 25 | 100.0% | 89 | 87.3% |
|  | Mild | 71 | 12.3% | 43 | 10.1% | 0 | 0.0% | 11 | 10.8% |
|  | Moderate | 42 | 7.3% | 12 | 2.8% | 0 | 0.0% | 2 | 2.0% |
|  | Severe | 17 | 2.9% | 4 | 0.9% | 0 | 0.0% | 0 | 0.0% |
| Shortness of breath | No side effect | 441 | 76.3% | 374 | 87.8% | 24 | 96.0% | 96 | 94.1% |
|  | Mild | 83 | 14.4% | 36 | 8.5% | 1 | 4.0% | 4 | 3.9% |
|  | Moderate | 37 | 6.4% | 10 | 2.3% | 0 | 0.0% | 1 | 1.0% |
|  | Severe | 17 | 2.9% | 6 | 1.4% | 0 | 0.0% | 1 | 1.0% |
| Vision disorders | No side effect | 441 | 76.3% | 376 | 88.3% | 23 | 92.0% | 93 | 91.2% |
|  | Mild | 88 | 15.2% | 33 | 7.7% | 2 | 8.0% | 6 | 5.9% |
|  | Moderate | 33 | 5.7% | 8 | 1.9% | 0 | 0.0% | 2 | 2.0% |
|  | Severe | 16 | 2.8% | 9 | 2.1% | 0 | 0.0% | 1 | 1.0% |
| Jaw pain | No side effect | 486 | 84.1% | 386 | 90.6% | 24 | 96.0% | 95 | 93.1% |
|  | Mild | 42 | 7.3% | 27 | 6.3% | 1 | 4.0% | 4 | 3.9% |
|  | Moderate | 24 | 4.2% | 9 | 2.1% | 0 | 0.0% | 2 | 2.0% |
|  | Severe | 26 | 4.5% | 4 | 0.9% | 0 | 0.0% | 1 | 1.0% |
| Earache | No side effect | 492 | 85.1% | 389 | 91.3% | 25 | 100.0% | 96 | 94.1% |
|  | Mild | 46 | 8.0% | 19 | 4.5% | 0 | 0.0% | 5 | 4.9% |
|  | Moderate | 18 | 3.1% | 12 | 2.8% | 0 | 0.0% | 0 | 0.0% |
|  | Severe | 22 | 3.8% | 6 | 1.4% | 0 | 0.0% | 1 | 1.0% |
| Vomiting | No side effect | 501 | 86.7% | 396 | 93.0% | 24 | 96.0% | 96 | 94.1% |
|  | Mild | 45 | 7.8% | 17 | 4.0% | 1 | 4.0% | 4 | 3.9% |
|  | Moderate | 12 | 2.1% | 8 | 1.9% | 0 | 0.0% | 1 | 1.0% |
|  | Severe | 20 | 3.5% | 5 | 1.2% | 0 | 0.0% | 1 | 1.0% |
| Cellulitis warmth and swollen armpit glands | No side effect | 517 | 89.4% | 397 | 93.2% | 24 | 96.0% | 95 | 93.1% |
|  | Mild | 45 | 7.8% | 21 | 4.9% | 1 | 4.0% | 7 | 6.9% |
|  | Moderate | 10 | 1.7% | 8 | 1.9% | 0 | 0.0% | 0 | 0.0% |
|  | Severe | 6 | 1.0% | 0 | 0.0% | 0 | 0.0% | 0 | 0.0% |
| Redness and itch | No side effect | 495 | 85.6% | 385 | 90.4% | 22 | 88.0% | 97 | 95.1% |
|  | Mild | 55 | 9.5% | 25 | 5.9% | 3 | 12.0% | 4 | 3.9% |
|  | Moderate | 21 | 3.6% | 13 | 3.1% | 0 | 0.0% | 0 | 0.0% |
|  | Severe | 7 | 1.2% | 3 | 0.7% | 0 | 0.0% | 1 | 1.0% |
| Blurred vision | No side effect | 479 | 82.9% | 389 | 91.3% | 25 | 100.0% | 99 | 97.1% |
|  | Mild | 58 | 10.0% | 25 | 5.9% | 0 | 0.0% | 2 | 2.0% |
|  | Moderate | 26 | 4.5% | 8 | 1.9% | 0 | 0.0% | 1 | 1.0% |
|  | Severe | 15 | 2.6% | 4 | 0.9% | 0 | 0.0% | 0 | 0.0% |
| Diplopia | No side effect | 530 | 91.7% | 408 | 95.8% | 25 | 100.0% | 98 | 96.1% |
|  | Mild | 34 | 5.9% | 13 | 3.1% | 0 | 0.0% | 3 | 2.9% |
|  | Moderate | 9 | 1.6% | 5 | 1.2% | 0 | 0.0% | 1 | 1.0% |
|  | Severe | 5 | 0.9% | 0 | 0.0% | 0 | 0.0% | 0 | 0.0% |
| Dysphagia | No side effect | 533 | 92.2% | 404 | 94.8% | 25 | 100.0% | 97 | 95.1% |
|  | Mild | 22 | 3.8% | 20 | 4.7% | 0 | 0.0% | 2 | 2.0% |
|  | Moderate | 16 | 2.8% | 2 | 0.5% | 0 | 0.0% | 2 | 2.0% |
|  | Severe | 7 | 1.2% | 0 | 0.0% | 0 | 0.0% | 1 | 1.0% |
| Facial numbness | No side effect | 569 | 98.4% | 415 | 97.4% | 25 | 100.0% | 102 | 100.0% |
|  | Mild | 8 | 1.4% | 11 | 2.6% | 0 | 0.0% | 0 | 0.0% |
|  | Moderate | 0 | 0.0% | 0 | 0.0% | 0 | 0.0% | 0 | 0.0% |
|  | Severe | 1 | 0.2% | 0 | 0.0% | 0 | 0.0% | 0 | 0.0% |
| Anesthesia (face/body) | No side effect | 537 | 92.9% | 402 | 94.4% | 23 | 92.0% | 93 | 91.2% |
|  | Mild | 23 | 4.0% | 17 | 4.0% | 1 | 4.0% | 7 | 6.9% |
|  | Moderate | 11 | 1.9% | 3 | 0.7% | 1 | 4.0% | 2 | 2.0% |
|  | Severe | 7 | 1.2% | 4 | 0.9% | 0 | 0.0% | 0 | 0.0% |
| Tachycardia | No side effect | 551 | 95.3% | 412 | 96.7% | 24 | 96.0% | 101 | 99.0% |
|  | Mild | 17 | 2.9% | 13 | 3.1% | 1 | 4.0% | 0 | 0.0% |
|  | Moderate | 7 | 1.2% | 1 | 0.2% | 0 | 0.0% | 0 | 0.0% |
|  | Severe | 3 | 0.5% | 0 | 0.0% | 0 | 0.0% | 1 | 1.0% |
| Anaphylactic shock | No side effect | 571 | 98.8% | 422 | 99.1% | 25 | 100.0% | 102 | 100.0% |
|  | Mild | 6 | 1.0% | 4 | 0.9% | 0 | 0.0% | 0 | 0.0% |
|  | Moderate | 1 | 0.2% | 0 | 0.0% | 0 | 0.0% | 0 | 0.0% |
|  | Severe | 0 | 0.0% | 0 | 0.0% | 0 | 0.0% | 0 | 0.0% |
| Thrombosis and blood-clotting | No side effect | 566 | 97.9% | 419 | 98.4% | 25 | 100.0% | 99 | 97.1% |
|  | Mild | 10 | 1.7% | 7 | 1.6% | 0 | 0.0% | 3 | 2.9% |
|  | Moderate | 1 | 0.2% | 0 | 0.0% | 0 | 0.0% | 0 | 0.0% |
|  | Severe | 1 | 0.2% | 0 | 0.0% | 0 | 0.0% | 0 | 0.0% |
| Oral bleeding | No side effect | 568 | 98.3% | 422 | 99.1% | 24 | 96.0% | 101 | 99.0% |
|  | Mild | 8 | 1.4% | 4 | 0.9% | 1 | 4.0% | 1 | 1.0% |
|  | Moderate | 2 | 0.3% | 0 | 0.0% | 0 | 0.0% | 0 | 0.0% |
|  | Severe | 0 | 0.0% | 0 | 0.0% | 0 | 0.0% | 0 | 0.0% |
| Nasal bleeding | No side effect | 557 | 96.4% | 412 | 96.7% | 24 | 96.0% | 101 | 99.0% |
|  | Mild | 17 | 2.9% | 12 | 2.8% | 1 | 4.0% | 1 | 1.0% |
|  | Moderate | 2 | 0.3% | 1 | 0.2% | 0 | 0.0% | 0 | 0.0% |
|  | Severe | 2 | 0.3% | 1 | 0.2% | 0 | 0.0% | 0 | 0.0% |
| Seizure | No side effect | 572 | 99.0% | 422 | 99.1% | 25 | 100.0% | 102 | 100.0% |
|  | Mild | 5 | 0.9% | 4 | 0.9% | 0 | 0.0% | 0 | 0.0% |
|  | Moderate | 0 | 0.0% | 0 | 0.0% | 0 | 0.0% | 0 | 0.0% |
|  | Severe | 1 | 0.2% | 0 | 0.0% | 0 | 0.0% | 0 | 0.0% |
| Faint | No side effect | 558 | 96.5% | 414 | 97.2% | 25 | 100.0% | 102 | 100.0% |
|  | Mild | 9 | 1.6% | 8 | 1.9% | 0 | 0.0% | 0 | 0.0% |
|  | Moderate | 7 | 1.2% | 2 | 0.5% | 0 | 0.0% | 0 | 0.0% |
|  | Severe | 4 | 0.7% | 2 | 0.5% | 0 | 0.0% | 0 | 0.0% |
| Optic neuritis | No side effect | 559 | 96.7% | 417 | 97.9% | 25 | 100.0% | 102 | 100.0% |
|  | Mild | 15 | 2.6% | 9 | 2.1% | 0 | 0.0% | 0 | 0.0% |
|  | Moderate | 3 | 0.5% | 0 | 0.0% | 0 | 0.0% | 0 | 0.0% |
|  | Severe | 1 | 0.2% | 0 | 0.0% | 0 | 0.0% | 0 | 0.0% |
| Speech disorders | No side effect | 556 | 96.2% | 415 | 97.4% | 25 | 100.0% | 101 | 99.0% |
|  | Mild | 19 | 3.3% | 9 | 2.1% | 0 | 0.0% | 1 | 1.0% |
|  | Moderate | 2 | 0.3% | 1 | 0.2% | 0 | 0.0% | 0 | 0.0% |
|  | Severe | 1 | 0.2% | 1 | 0.2% | 0 | 0.0% | 0 | 0.0% |

Table. The severity reported (percentage) for each side effect based on the type of vaccine.

| Onset of vaccine side effects: | Astrazeneca | Sputnic V | Covaxine | Sinopharm | **P-value** |
| --- | --- | --- | --- | --- | --- |
| 0-12 | 304(52.6%) | 150(35.2%) | 9(36.0%) | 36(35.3%) | <0.001 |
| 12-24 | 196(33.9%) | 113(26.5%) | 7(28.0%) | 12(11.8%) |  |
| 1-2 | 31(5.4%) | 52(12.2%) | 2(8.0%) | 11(10.8%) |  |
| 3-4 | 7(1.2%) | 11(2.6%) | 0(0.0%) | 1(1.0%) |  |
| 5-7 | 0(0.0%) | 6(1.4%) | 1(4.0%) | 2(2.0%) |  |
| 1-2 week | 2(0.3%) | 8(1.9%) | 1(4.0%) | 1(1.0%) |  |
| 3-4week | 1(0.2%) | 1(0.2%) | 0(0.0%) | 1(1.0%) |  |
| No side effect | 37(6.4%) | 85(20.0%) | 5(20.0%) | 38(37.3%) |  |

Table. The onset of symptoms based on type of the administered vaccine.

|  | Astrazeneca | Sputnic V | Covaxine | Sinopharm | **Total** | **P-value** |
| --- | --- | --- | --- | --- | --- | --- |
| Fatigue | 6±3 | 3±3 | 3±3 | 2±3 | 4±3 | **<0.001** |
| Malaise | 5±3 | 3±3 | 2±3 | 1±1 | 4±3 | **<0.001** |
| headache | 4±4 | 2±3 | 2±3 | 2±3 | 3±4 | **<0.001** |
| Body pain | 6±4 | 3±3 | 3±3 | 1±2 | 4±4 | **<0.001** |
| Visual disorder | 1±2 | 0±1 | 0±0 | 0±1 | 1±2 | **<0.001** |
| vomiting | 1±2 | 0±1 | 0±0 | 0±1 | 0±2 | **0.003** |
| fever | 5±4 | 2±3 | 1±2 | 1±1 | 3±4 | **<0.001** |
| chills and shiver | 4±4 | 2±3 | 1±3 | 0±1 | 3±4 | **<0.001** |
| local pain in hand | 5±3 | 3±3 | 5±3 | 3±3 | 4±3 | **<0.001** |
| pain in foot | 3±4 | 2±3 | 2±3 | 1±2 | 2±3 | **<0.001** |
| cellulitis warmth and swollen armpit glands | 0±1 | 0±1 | 0±0 | 0±0 | 0±1 | 0.117 |
| loss of appetite | 2±3 | 1±2 | 1±2 | 0±1 | 2±3 | **<0.001** |
| dizziness | 2±3 | 1±2 | 1±1 | 1±1 | 2±3 | **<0.001** |
| redness and itch | 0±1 | 0±1 | 0±0 | 0±1 | 0±1 | **0.015** |
| Crump/ abdominal pain | 1±2 | 0±1 | 1±2 | 0±1 | 1±2 | **<0.001** |
| arthralgia | 3±4 | 2±3 | 2±3 | 1±1 | 2±3 | **<0.001** |
| chest pain | 1±2 | 1±2 | 0±1 | 0±1 | 1±2 | **<0.001** |
| cough | 1±2 | 0±1 | 0±0 | 0±1 | 1±2 | **<0.001** |
| shortness of breath | 1±2 | 0±1 | 0±0 | 0±1 | 1±2 | **<0.001** |
| Blurred vision | 1±2 | 0±1 | 0±0 | 0±0 | 0±1 | **<0.001** |
| Diplopia | 0±1 | 0±1 | 0±0 | 0±0 | 0±1 | **0.020** |
| diarrhea | 1±2 | 0±1 | 1±2 | 0±0 | 1±2 | **<0.001** |
| insomnia | 2±3 | 1±2 | 0±1 | 0±1 | 2±3 | **<0.001** |
| jaw pain | 1±2 | 0±1 | 0±0 | 0±1 | 0±2 | **0.001** |
| Earache | 1±2 | 0±1 | 0±0 | 0±1 | 0±2 | **0.001** |
| dysphagia | 0±1 | 0±1 | 0±0 | 0±1 | 0±1 | 0.147 |
| facial numbness | 0±0 | 0±0 | 0±0 | 0±0 | 0±0 | 0.258 |
| anesthesia (face/body) | 0±1 | 0±1 | 0±1 | 0±1 | 0±1 | 0.644 |
| bradycardia | 2±3 | 1±2 | 0±1 | 0±1 | 1±2 | **<0.001** |
| tachycardia | 0±1 | 0±0 | 0±0 | 0±1 | 0±1 | 0.288 |
| Anaphylactic shock | 0±0 | 0±0 | 0±0 | 0±0 | 0±0 | 0.661 |
| thrombosis and blood-clotting | 0±0 | 0±0 | 0±0 | 0±0 | 0±0 | 0.733 |
| Oral bleeding | 0±0 | 0±0 | 0±0 | 0±0 | 0±0 | 0.486 |
| Nasal bleeding | 0±1 | 0±1 | 0±0 | 0±0 | 0±1 | 0.575 |
| Seizure | 0±0 | 0±0 | 0±0 | 0±0 | 0±0 | 0.728 |
| Faint | 0±1 | 0±1 | 0±0 | 0±0 | 0±1 | 0.207 |
| Optic neuritis | 0±1 | 0±0 | 0±0 | 0±0 | 0±0 | 0.168 |
| Speech disorders | 0±1 | 0±1 | 0±0 | 0±0 | 0±1 | 0.284 |

|  | | Influenza | | | | MERS | | | | EBV | | | | No history of viral infection | | | |
| --- | --- | --- | --- | --- | --- | --- | --- | --- | --- | --- | --- | --- | --- | --- | --- | --- | --- |
|  | | Astrazeneca | Sputnic V | Covaxine | Sinopharm | Astrazeneca | Sputnic V | Covaxine | Sinopharm | Astrazeneca | Sputnic V | Covaxine | Sinopharm | Astrazeneca | Sputnic V | Covaxine | Sinopharm |
| 0-12 | N | 92 | 47 | 2 | 11 | 1 | 0 | 0 | 0 | 6 | 3 | 0 | 2 | 205 | 100 | 7 | 23 |
|  | % | 58.6% | 37.3% | 33.3% | 37.9% | 100.0% | 0.0% | 0.0% | 0.0% | 54.5% | 75.0% | 0.0% | 100.0% | 50.1% | 33.8% | 36.8% | 32.4% |
| 12-24 | N | 49 | 33 | 1 | 3 | 0 | 0 | 0 | 0 | 3 | 1 | 0 | 0 | 144 | 79 | 6 | 9 |
|  | % | 31.2% | 26.2% | 16.7% | 10.3% | 0.0% | 0.0% | 0.0% | 0.0% | 27.3% | 25.0% | 0.0% | 0.0% | 35.2% | 26.7% | 31.6% | 12.7% |
| 1-2 | N | 5 | 21 | 1 | 4 | 0 | 0 | 0 | 0 | 0 | 0 | 0 | 0 | 26 | 31 | 1 | 7 |
|  | % | 3.2% | 16.7% | 16.7% | 13.8% | 0.0% | 0.0% | 0.0% | 0.0% | 0.0% | 0.0% | 0.0% | 0.0% | 6.4% | 10.5% | 5.3% | 9.9% |
| 3-4 | N | 1 | 3 | 0 | 1 | 0 | 0 | 0 | 0 | 1 | 0 | 0 | 0 | 5 | 8 | 0 | 0 |
|  | % | 0.6% | 2.4% | 0.0% | 3.4% | 0.0% | 0.0% | 0.0% | 0.0% | 9.1% | 0.0% | 0.0% | 0.0% | 1.2% | 2.7% | 0.0% | 0.0% |
| 5-7 | N | 0 | 1 | 0 | 0 | 0 | 0 | 0 | 0 | 0 | 0 | 0 | 0 | 0 | 5 | 1 | 2 |
|  | % | 0.0% | 0.8% | 0.0% | 0.0% | 0.0% | 0.0% | 0.0% | 0.0% | 0.0% | 0.0% | 0.0% | 0.0% | 0.0% | 1.7% | 5.3% | 2.8% |
| 1-2 W | N | 1 | 2 | 0 | 1 | 0 | 0 | 0 | 0 | 0 | 0 | 0 | 0 | 1 | 6 | 1 | 0 |
|  | % | 0.6% | 1.6% | 0.0% | 3.4% | 0.0% | 0.0% | 0.0% | 0.0% | 0.0% | 0.0% | 0.0% | 0.0% | 0.2% | 2.0% | 5.3% | 0.0% |
| 3-4 W | N | 0 | 1 | 0 | 0 | 0 | 0 | 0 | 0 | 0 | 0 | 0 | 0 | 1 | 0 | 0 | 1 |
|  | % | 0.0% | 0.8% | 0.0% | 0.0% | 0.0% | 0.0% | 0.0% | 0.0% | 0.0% | 0.0% | 0.0% | 0.0% | 0.2% | 0.0% | 0.0% | 1.4% |
| No symptoms | N | 9 | 18 | 2 | 9 | 0 | 0 | 0 | 0 | 1 | 0 | 0 | 0 | 27 | 67 | 3 | 29 |
|  | % | 5.7% | 14.3% | 33.3% | 31.0% | 0.0% | 0.0% | 0.0% | 0.0% | 9.1% | 0.0% | 0.0% | 0.0% | 6.6% | 22.6% | 15.8% | 40.8% |
| **P-value** | | <0.001 | | | | NA | | | | 0.895 | | | | <0.001 | | | |

Table. Data regarding the mean and standard deviation of different symptoms in total and based on type of the administered vaccine.

|  | | Females( number: 681) | | | | Males (number: 451) | | | |
| --- | --- | --- | --- | --- | --- | --- | --- | --- | --- |
|  | | Astrazeneca | Sputnic V | Covaxine | Sinopharm | Astrazeneca | Sputnic V | Covaxine | Sinopharm |
| Fatigue | Mean | 6 | 4 | 3 | 2 | 5 | 2 | 3 | 2 |
|  | Standard Deviation | 3 | 3 | 3 | 3 | 3 | 3 | 3 | 2 |
| Malaise | Mean | 6 | 4 | 2 | 2 | 5 | 2 | 2 | 1 |
|  | Standard Deviation | 3 | 3 | 3 | 2 | 3 | 2 | 3 | 2 |
| headache | Mean | 5 | 3 | 2 | 2 | 4 | 1 | 2 | 1 |
|  | Standard Deviation | 4 | 3 | 2 | 3 | 3 | 3 | 3 | 3 |
| Body pain | Mean | 6 | 4 | 3 | 1 | 5 | 2 | 2 | 1 |
|  | Standard Deviation | 3 | 4 | 3 | 2 | 4 | 3 | 4 | 2 |
| Vision disorder | Mean | 1 | 1 | 0 | 0 | 1 | 0 | 0 | 0 |
|  | Standard Deviation | 2 | 2 | 0 | 1 | 2 | 1 | 0 | 0 |
| vomiting | Mean | 1 | 0 | 0 | 0 | 1 | 0 | 0 | 0 |
|  | Standard Deviation | 2 | 2 | 0 | 1 | 2 | 1 | 0 | 1 |
| fever | Mean | 5 | 3 | 0 | 1 | 4 | 1 | 2 | 0 |
|  | Standard Deviation | 4 | 3 | 1 | 1 | 4 | 2 | 3 | 1 |
| chills and shiver | Mean | 5 | 3 | 1 | 1 | 3 | 1 | 1 | 0 |
|  | Standard Deviation | 4 | 3 | 2 | 2 | 4 | 2 | 3 | 0 |
| local pain in hand | Mean | 5 | 4 | 6 | 3 | 4 | 2 | 3 | 2 |
|  | Standard Deviation | 3 | 3 | 3 | 3 | 3 | 3 | 3 | 2 |
| pain in foot | Mean | 4 | 2 | 3 | 1 | 2 | 1 | 1 | 1 |
|  | Standard Deviation | 4 | 3 | 4 | 2 | 3 | 2 | 2 | 2 |
| cellulitis warmth and swollen armpit glands | Mean | 0 | 0 | 0 | 0 | 0 | 0 | 0 | 0 |
|  | Standard Deviation | 1 | 1 | 0 | 0 | 1 | 1 | 0 | 0 |
| loss of appetite | Mean | 3 | 1 | 1 | 1 | 2 | 0 | 0 | 0 |
|  | Standard Deviation | 3 | 2 | 3 | 1 | 3 | 1 | 1 | 1 |
| dizziness | Mean | 3 | 1 | 1 | 1 | 2 | 1 | 1 | 1 |
|  | Standard Deviation | 3 | 2 | 2 | 1 | 3 | 2 | 1 | 2 |
| redness and itch | Mean | 0 | 0 | 0 | 0 | 0 | 0 | 0 | 0 |
|  | Standard Deviation | 1 | 1 | 0 | 1 | 1 | 1 | 0 | 0 |
| Crump/ abdominal pain | Mean | 1 | 1 | 1 | 0 | 1 | 0 | 1 | 0 |
|  | Standard Deviation | 2 | 2 | 2 | 1 | 2 | 1 | 2 | 0 |
| arthralgia | Mean | 3 | 3 | 2 | 1 | 2 | 1 | 1 | 0 |
|  | Standard Deviation | 4 | 3 | 3 | 2 | 3 | 2 | 2 | 1 |
| chest pain | Mean | 1 | 1 | 1 | 0 | 1 | 0 | 0 | 0 |
|  | Standard Deviation | 3 | 2 | 2 | 1 | 2 | 1 | 1 | 1 |
| Cough | Mean | 1 | 0 | 0 | 0 | 1 | 0 | 0 | 0 |
|  | Standard Deviation | 2 | 1 | 0 | 1 | 1 | 1 | 0 | 0 |
| shortness of breath | Mean | 1 | 0 | 0 | 0 | 1 | 0 | 0 | 0 |
|  | Standard Deviation | 2 | 2 | 0 | 1 | 2 | 1 | 0 | 2 |
| Blurred vision | Mean | 1 | 0 | 0 | 0 | 0 | 0 | 0 | 0 |
|  | Standard Deviation | 2 | 1 | 0 | 0 | 1 | 1 | 0 | 0 |
| Diplopia | Mean | 0 | 0 | 0 | 0 | 0 | 0 | 0 | 0 |
|  | Standard Deviation | 1 | 1 | 0 | 1 | 1 | 1 | 0 | 0 |
| diarrhea | Mean | 1 | 0 | 1 | 0 | 1 | 0 | 0 | 0 |
|  | Standard Deviation | 2 | 1 | 3 | 1 | 2 | 1 | 0 | 1 |
|  | Standard Deviation | 3 | 4 | 3 | 2 | 3 | 3 | 3 | 2 |
| insomnia | Mean | 3 | 2 | 1 | 1 | 2 | 1 | 0 | 0 |
|  | Standard Deviation | 3 | 3 | 2 | 1 | 3 | 2 | 0 | 1 |
| jaw pain | Mean | 1 | 0 | 0 | 0 | 0 | 0 | 0 | 0 |
|  | Standard Deviation | 2 | 1 | 0 | 1 | 1 | 1 | 0 | 2 |
| Earache | Mean | 1 | 0 | 0 | 0 | 0 | 0 | 0 | 0 |
|  | Standard Deviation | 2 | 2 | 0 | 1 | 1 | 1 | 0 | 0 |
| dysphagia | Mean | 0 | 0 | 0 | 0 | 0 | 0 | 0 | 0 |
|  | Standard Deviation | 1 | 1 | 0 | 1 | 1 | 0 | 0 | 2 |
| facial numbness | Mean | 0 | 0 | 0 | 0 | 0 | 0 | 0 | 0 |
|  | Standard Deviation | 0 | 0 | 0 | 0 | 1 | 0 | 0 | 0 |
| anesthesia (face/body) | Mean | 0 | 0 | 0 | 0 | 0 | 0 | 0 | 0 |
|  | Standard Deviation | 1 | 1 | 0 | 1 | 1 | 1 | 1 | 0 |
| Bradycardia | Mean | 2 | 1 | 0 | 1 | 1 | 1 | 0 | 0 |
|  | Standard Deviation | 3 | 2 | 1 | 1 | 2 | 2 | 0 | 1 |
| Tachycardia | Mean | 0 | 0 | 0 | 0 | 0 | 0 | 0 | 0 |
|  | Standard Deviation | 1 | 0 | 0 | 1 | 1 | 0 | 0 | 0 |
| Anaphylactic shock | Mean | 0 | 0 | 0 | 0 | 0 | 0 | 0 | 0 |
|  | Standard Deviation | 0 | 0 | 0 | 0 | 0 | 0 | 0 | 0 |
| thrombosis and blood-clotting | Mean | 0 | 0 | 0 | 0 | 0 | 0 | 0 | 0 |
|  | Standard Deviation | 0 | 0 | 0 | 0 | 1 | 0 | 0 | 0 |
| Oral bleeding | Mean | 0 | 0 | 0 | 0 | 0 | 0 | 0 | 0 |
|  | Standard Deviation | 0 | 0 | 0 | 0 | 0 | 0 | 0 | 0 |
| Nasal bleeding | Mean | 0 | 0 | 0 | 0 | 0 | 0 | 0 | 0 |
|  | Standard Deviation | 1 | 1 | 0 | 0 | 0 | 0 | 0 | 0 |
| Seizure | Mean | 0 | 0 | 0 | 0 | 0 | 0 | 0 | 0 |
|  | Standard Deviation | 1 | 0 | 0 | 0 | 0 | 0 | 0 | 0 |
| Faint | Mean | 0 | 0 | 0 | 0 | 0 | 0 | 0 | 0 |
|  | Standard Deviation | 1 | 1 | 0 | 0 | 1 | 1 | 0 | 0 |
| Optic neuritis | Mean | 0 | 0 | 0 | 0 | 0 | 0 | 0 | 0 |
|  | Standard Deviation | 0 | 0 | 0 | 0 | 1 | 0 | 0 | 0 |
| Speech disorders | Mean | 0 | 0 | 0 | 0 | 0 | 0 | 0 | 0 |
|  | Standard Deviation | 1 | 0 | 0 | 0 | 0 | 1 | 0 | 0 |

Table X illustrates the mean and standard deviation of symptoms in males and females based on the type of vaccine used.

|  | | Under 40 (number: 675) | | | | 40 and higher (number: 420) | | | |
| --- | --- | --- | --- | --- | --- | --- | --- | --- | --- |
|  | | Astrazeneca | Sputnic V | Covaxine | Sinopharm | Astrazeneca | Sputnic V | Covaxine | Sinopharm |
| Fatigue | Mean | 6 | 5 | 3 | 2 | 5 | 2 | 4 | 2 |
|  | Standard Deviation | 3 | 3 | 3 | 3 | 3 | 3 | 4 | 2 |
| Malaise | Mean | 5 | 4 | 2 | 1 | 4 | 2 | 3 | 1 |
|  | Standard Deviation | 3 | 3 | 3 | 2 | 3 | 3 | 3 | 2 |
| headache | Mean | 5 | 3 | 2 | 2 | 4 | 2 | 2 | 1 |
|  | Standard Deviation | 4 | 3 | 3 | 3 | 3 | 3 | 3 | 2 |
| Body pain | Mean | 6 | 5 | 3 | 1 | 4 | 2 | 2 | 1 |
|  | Standard Deviation | 4 | 4 | 3 | 2 | 3 | 3 | 4 | 2 |
| Vision disorder | Mean | 1 | 1 | 0 | 0 | 1 | 0 | 0 | 0 |
|  | Standard Deviation | 2 | 2 | 0 | 1 | 2 | 1 | 0 | 0 |
| vomiting | Mean | 1 | 0 | 0 | 0 | 0 | 0 | 0 | 0 |
|  | Standard Deviation | 2 | 2 | 0 | 1 | 1 | 1 | 0 | 0 |
| fever | Mean | 5 | 3 | 1 | 1 | 3 | 1 | 1 | 0 |
|  | Standard Deviation | 4 | 3 | 1 | 1 | 3 | 2 | 3 | 1 |
| chills and shiver | Mean | 5 | 4 | 1 | 1 | 3 | 1 | 1 | 0 |
|  | Standard Deviation | 4 | 4 | 2 | 1 | 4 | 2 | 3 | 1 |
| local pain in hand | Mean | 5 | 5 | 6 | 3 | 4 | 3 | 3 | 1 |
|  | Standard Deviation | 3 | 4 | 3 | 3 | 4 | 3 | 2 | 2 |
| pain in foot | Mean | 3 | 3 | 2 | 1 | 2 | 1 | 1 | 1 |
|  | Standard Deviation | 4 | 4 | 4 | 2 | 3 | 2 | 2 | 2 |
| cellulitis warmth and swollen armpit glands | Mean | 0 | 0 | 0 | 0 | 0 | 0 | 0 | 0 |
|  | Standard Deviation | 1 | 1 | 0 | 0 | 1 | 1 | 0 | 0 |
| loss of appetite | Mean | 3 | 1 | 1 | 1 | 1 | 0 | 0 | 0 |
|  | Standard Deviation | 3 | 2 | 2 | 1 | 2 | 1 | 1 | 1 |
| dizziness | Mean | 3 | 2 | 1 | 1 | 2 | 1 | 1 | 0 |
|  | Standard Deviation | 3 | 3 | 1 | 2 | 3 | 2 | 1 | 0 |
| redness and itch | Mean | 1 | 0 | 0 | 0 | 0 | 0 | 0 | 0 |
|  | Standard Deviation | 2 | 1 | 0 | 1 | 1 | 1 | 0 | 0 |
| Crump/ abdominal pain | Mean | 1 | 1 | 1 | 0 | 1 | 0 | 0 | 0 |
|  | Standard Deviation | 2 | 2 | 3 | 1 | 2 | 1 | 1 | 0 |
| arthralgia | Mean | 3 | 3 | 2 | 1 | 2 | 1 | 1 | 1 |
|  | Standard Deviation | 4 | 4 | 3 | 2 | 3 | 2 | 2 | 1 |
| chest pain | Mean | 1 | 1 | 1 | 0 | 1 | 0 | 0 | 0 |
|  | Standard Deviation | 3 | 2 | 1 | 1 | 2 | 1 | 0 | 0 |
| Cough | Mean | 1 | 1 | 0 | 0 | 0 | 0 | 0 | 0 |
|  | Standard Deviation | 2 | 2 | 0 | 1 | 1 | 1 | 0 | 0 |
| shortness of breath | Mean | 1 | 1 | 0 | 0 | 1 | 0 | 0 | 0 |
|  | Standard Deviation | 2 | 2 | 0 | 1 | 2 | 1 | 0 | 0 |
| Blurred vision | Mean | 1 | 0 | 0 | 0 | 1 | 0 | 0 | 0 |
|  | Standard Deviation | 2 | 1 | 0 | 0 | 2 | 1 | 0 | 0 |
| Diplopia | Mean | 0 | 0 | 0 | 0 | 0 | 0 | 0 | 0 |
|  | Standard Deviation | 1 | 1 | 0 | 1 | 0 | 1 | 0 | 0 |
| diarrhea | Mean | 1 | 0 | 1 | 0 | 0 | 0 | 0 | 0 |
|  | Standard Deviation | 2 | 2 | 3 | 1 | 1 | 1 | 0 | 0 |
|  | Standard Deviation | 3 | 3 | 3 | 2 | 3 | 3 | 3 | 2 |
| insomnia | Mean | 2 | 2 | 0 | 0 | 2 | 1 | 1 | 1 |
|  | Standard Deviation | 3 | 3 | 0 | 1 | 3 | 2 | 2 | 1 |
| jaw pain | Mean | 1 | 0 | 0 | 0 | 0 | 0 | 0 | 0 |
|  | Standard Deviation | 2 | 2 | 0 | 1 | 2 | 1 | 0 | 0 |
| Earache | Mean | 1 | 1 | 0 | 0 | 0 | 0 | 0 | 0 |
|  | Standard Deviation | 2 | 2 | 0 | 1 | 1 | 1 | 0 | 0 |
| dysphagia | Mean | 0 | 0 | 0 | 0 | 0 | 0 | 0 | 0 |
|  | Standard Deviation | 1 | 1 | 0 | 1 | 1 | 0 | 0 | 0 |
| facial numbness | Mean | 0 | 0 | 0 | 0 | 0 | 0 | 0 | 0 |
|  | Standard Deviation | 0 | 0 | 0 | 0 | 0 | 0 | 0 | 0 |
| anesthesia (face/body) | Mean | 0 | 0 | 0 | 0 | 0 | 0 | 0 | 0 |
|  | Standard Deviation | 1 | 1 | 0 | 1 | 0 | 1 | 1 | 0 |
| Bradycardia | Mean | 2 | 1 | 0 | 1 | 1 | 1 | 0 | 0 |
|  | Standard Deviation | 3 | 3 | 1 | 2 | 2 | 2 | 0 | 0 |
| Tachycardia | Mean | 0 | 0 | 0 | 0 | 0 | 0 | 0 | 0 |
|  | Standard Deviation | 1 | 0 | 0 | 1 | 0 | 0 | 0 | 0 |
| Anaphylactic shock | Mean | 0 | 0 | 0 | 0 | 0 | 0 | 0 | 0 |
|  | Standard Deviation | 0 | 0 | 0 | 0 | 0 | 0 | 0 | 0 |
| thrombosis and blood-clotting | Mean | 0 | 0 | 0 | 0 | 0 | 0 | 0 | 0 |
|  | Standard Deviation | 1 | 0 | 0 | 0 | 0 | 0 | 0 | 0 |
| Oral bleeding | Mean | 0 | 0 | 0 | 0 | 0 | 0 | 0 | 0 |
|  | Standard Deviation | 0 | 0 | 0 | 0 | 0 | 0 | 0 | 0 |
| Nasal bleeding | Mean | 0 | 0 | 0 | 0 | 0 | 0 | 0 | 0 |
|  | Standard Deviation | 1 | 1 | 0 | 0 | 0 | 0 | 0 | 0 |
| Seizure | Mean | 0 | 0 | 0 | 0 | 0 | 0 | 0 | 0 |
|  | Standard Deviation | 0 | 0 | 0 | 0 | 0 | 0 | 0 | 0 |
| Faint | Mean | 0 | 0 | 0 | 0 | 0 | 0 | 0 | 0 |
|  | Standard Deviation | 1 | 1 | 0 | 0 | 1 | 1 | 0 | 0 |
| Optic neuritis | Mean | 0 | 0 | 0 | 0 | 0 | 0 | 0 | 0 |
|  | Standard Deviation | 1 | 0 | 0 | 0 | 0 | 0 | 0 | 0 |
| Speech disorders | Mean | 0 | 0 | 0 | 0 | 0 | 0 | 0 | 0 |
|  | Standard Deviation | 0 | 1 | 0 | 0 | 0 | 0 | 0 | 0 |

Table. The mean and standard deviation of symptoms in two age groups (under 40 and above 40) based on the type of vaccine used.

| History of COVID | | No | | | | Yes | | | |
| --- | --- | --- | --- | --- | --- | --- | --- | --- | --- |
|  | | Astrazeneca | Sputnic V | Covaxine | Sinopharm | Astrazeneca | Sputnic V | Covaxine | Sinopharm |
| Fatigue | Mean | 6 | 3 | 3 | 2 | 6 | 4 | 3 | 3 |
|  | Standard Deviation | 3 | 3 | 3 | 2 | 3 | 3 | 3 | 3 |
| Malaise | Mean | 5 | 3 | 2 | 1 | 5 | 3 | 2 | 2 |
|  | Standard Deviation | 3 | 3 | 3 | 2 | 3 | 3 | 3 | 3 |
| headache | Mean | 4 | 2 | 2 | 2 | 5 | 3 | 2 | 1 |
|  | Standard Deviation | 4 | 3 | 3 | 3 | 4 | 3 | 2 | 3 |
| Body pain | Mean | 6 | 3 | 3 | 1 | 6 | 4 | 2 | 1 |
|  | Standard Deviation | 4 | 3 | 4 | 2 | 4 | 4 | 3 | 2 |
| Vision disorder | Mean | 1 | 0 | 0 | 0 | 1 | 0 | 0 | 0 |
|  | Standard Deviation | 2 | 1 | 0 | 1 | 2 | 1 | 0 | 1 |
| vomiting | Mean | 1 | 0 | 0 | 0 | 1 | 0 | 0 | 0 |
|  | Standard Deviation | 2 | 1 | 0 | 1 | 2 | 1 | 0 | 0 |
| fever | Mean | 4 | 2 | 1 | 0 | 5 | 3 | 1 | 1 |
|  | Standard Deviation | 4 | 3 | 2 | 1 | 4 | 3 | 2 | 1 |
| chills and shiver | Mean | 4 | 2 | 1 | 0 | 5 | 3 | 1 | 1 |
|  | Standard Deviation | 4 | 3 | 3 | 1 | 4 | 4 | 2 | 1 |
| local pain in hand | Mean | 5 | 3 | 5 | 3 | 5 | 4 | 6 | 3 |
|  | Standard Deviation | 3 | 3 | 3 | 3 | 3 | 3 | 3 | 3 |
| pain in foot | Mean | 3 | 2 | 3 | 1 | 3 | 2 | 0 | 1 |
|  | Standard Deviation | 4 | 3 | 4 | 2 | 4 | 3 | 0 | 3 |
| cellulitis warmth and swollen armpit glands | Mean | 0 | 0 | 0 | 0 | 0 | 0 | 0 | 0 |
|  | Standard Deviation | 1 | 1 | 0 | 0 | 1 | 1 | 0 | 0 |
| loss of appetite | Mean | 2 | 1 | 1 | 0 | 3 | 1 | 1 | 1 |
|  | Standard Deviation | 3 | 2 | 2 | 1 | 3 | 2 | 1 | 1 |
| dizziness | Mean | 2 | 1 | 1 | 1 | 3 | 1 | 1 | 0 |
|  | Standard Deviation | 3 | 2 | 1 | 2 | 3 | 2 | 1 | 1 |
| redness and itch | Mean | 0 | 0 | 0 | 0 | 1 | 0 | 0 | 0 |
|  | Standard Deviation | 1 | 1 | 0 | 1 | 1 | 2 | 0 | 0 |
| Crump/ abdominal pain | Mean | 1 | 0 | 1 | 0 | 1 | 1 | 1 | 0 |
|  | Standard Deviation | 2 | 1 | 2 | 1 | 2 | 2 | 2 | 1 |
| arthralgia | Mean | 3 | 2 | 2 | 1 | 4 | 2 | 1 | 1 |
|  | Standard Deviation | 3 | 3 | 3 | 1 | 4 | 3 | 2 | 2 |
| chest pain | Mean | 1 | 1 | 1 | 0 | 2 | 1 | 0 | 0 |
|  | Standard Deviation | 2 | 2 | 1 | 1 | 3 | 2 | 1 | 1 |
| Cough | Mean | 1 | 0 | 0 | 0 | 1 | 0 | 0 | 0 |
|  | Standard Deviation | 2 | 1 | 0 | 0 | 2 | 1 | 0 | 1 |
| shortness of breath | Mean | 1 | 0 | 0 | 0 | 1 | 1 | 0 | 0 |
|  | Standard Deviation | 2 | 1 | 0 | 1 | 2 | 2 | 0 | 0 |
| Blurred vision | Mean | 1 | 0 | 0 | 0 | 1 | 0 | 0 | 0 |
|  | Standard Deviation | 2 | 1 | 0 | 0 | 2 | 1 | 0 | 0 |
| Diplopia | Mean | 0 | 0 | 0 | 0 | 0 | 0 | 0 | 0 |
|  | Standard Deviation | 1 | 1 | 0 | 0 | 1 | 0 | 0 | 0 |
| diarrhea | Mean | 1 | 0 | 1 | 0 | 1 | 0 | 0 | 0 |
|  | Standard Deviation | 2 | 1 | 3 | 1 | 2 | 1 | 0 | 2 |
|  | Standard Deviation | 3 | 3 | 3 | 2 | 4 | 4 | 2 | 2 |
| insomnia | Mean | 2 | 1 | 0 | 0 | 3 | 2 | 0 | 1 |
|  | Standard Deviation | 3 | 2 | 1 | 1 | 3 | 3 | 0 | 1 |
| jaw pain | Mean | 1 | 0 | 0 | 0 | 1 | 0 | 0 | 0 |
|  | Standard Deviation | 2 | 1 | 0 | 1 | 2 | 1 | 0 | 1 |
| Earache | Mean | 1 | 0 | 0 | 0 | 1 | 0 | 0 | 0 |
|  | Standard Deviation | 2 | 1 | 0 | 1 | 2 | 1 | 0 | 1 |
| dysphagia | Mean | 0 | 0 | 0 | 0 | 0 | 0 | 0 | 0 |
|  | Standard Deviation | 1 | 0 | 0 | 1 | 1 | 1 | 0 | 0 |
| facial numbness | Mean | 0 | 0 | 0 | 0 | 0 | 0 | 0 | 0 |
|  | Standard Deviation | 0 | 0 | 0 | 0 | 0 | 0 | 0 | 0 |
| anesthesia (face/body) | Mean | 0 | 0 | 0 | 0 | 0 | 0 | 0 | 0 |
|  | Standard Deviation | 1 | 1 | 1 | 1 | 1 | 1 | 0 | 0 |
| Bradycardia | Mean | 2 | 1 | 0 | 0 | 2 | 1 | 0 | 1 |
|  | Standard Deviation | 3 | 2 | 1 | 1 | 3 | 2 | 1 | 1 |
| Tachycardia | Mean | 0 | 0 | 0 | 0 | 0 | 0 | 0 | 0 |
|  | Standard Deviation | 1 | 0 | 0 | 0 | 1 | 0 | 0 | 1 |
| Anaphylactic shock | Mean | 0 | 0 | 0 | 0 | 0 | 0 | 0 | 0 |
|  | Standard Deviation | 0 | 0 | 0 | 0 | 1 | 0 | 0 | 0 |
| thrombosis and blood-clotting | Mean | 0 | 0 | 0 | 0 | 0 | 0 | 0 | 0 |
|  | Standard Deviation | 1 | 0 | 0 | 0 | 0 | 0 | 0 | 0 |
| Oral bleeding | Mean | 0 | 0 | 0 | 0 | 0 | 0 | 0 | 0 |
|  | Standard Deviation | 0 | 0 | 0 | 0 | 0 | 0 | 0 | 0 |
| Nasal bleeding | Mean | 0 | 0 | 0 | 0 | 0 | 0 | 0 | 0 |
|  | Standard Deviation | 0 | 1 | 0 | 0 | 1 | 0 | 0 | 0 |
| Seizure | Mean | 0 | 0 | 0 | 0 | 0 | 0 | 0 | 0 |
|  | Standard Deviation | 0 | 0 | 0 | 0 | 0 | 0 | 0 | 0 |
| Faint | Mean | 0 | 0 | 0 | 0 | 0 | 0 | 0 | 0 |
|  | Standard Deviation | 1 | 1 | 0 | 0 | 1 | 1 | 0 | 0 |
| Optic neuritis | Mean | 0 | 0 | 0 | 0 | 0 | 0 | 0 | 0 |
|  | Standard Deviation | 1 | 0 | 0 | 0 | 0 | 0 | 0 | 0 |
| Speech disorders | Mean | 0 | 0 | 0 | 0 | 0 | 0 | 0 | 0 |
|  | Standard Deviation | 1 | 1 | 0 | 0 | 1 | 1 | 0 | 0 |

Table. the mean and standard deviation of symptoms in patients with/without a history of covid infection (based on the type of vaccine used.

|  |  | No | | | | Yes | | | |
| --- | --- | --- | --- | --- | --- | --- | --- | --- | --- |
| History of other infections |  | Astrazeneca | Sputnic V | Covaxine | Sinopharm | Astrazeneca | Sputnic V | Covaxine | Sinopharm |
| Fatigue | Mean | 5 | 3 | 3 | 2 | 6 | 4 | 3 | 2 |
|  | Standard Deviation | 3 | 3 | 3 | 3 | 3 | 3 | 2 | 3 |
| Malaise | Mean | 5 | 3 | 3 | 1 | 6 | 3 | 2 | 1 |
|  | Standard Deviation | 3 | 3 | 3 | 2 | 3 | 3 | 2 | 3 |
| headache | Mean | 4 | 2 | 2 | 2 | 5 | 3 | 1 | 2 |
|  | Standard Deviation | 4 | 3 | 3 | 3 | 4 | 3 | 2 | 3 |
| Body pain | Mean | 6 | 3 | 2 | 1 | 6 | 3 | 3 | 1 |
|  | Standard Deviation | 4 | 3 | 4 | 2 | 4 | 3 | 3 | 2 |
| Vision disorder | Mean | 1 | 0 | 0 | 0 | 1 | 0 | 0 | 0 |
|  | Standard Deviation | 2 | 1 | 0 | 1 | 2 | 1 | 0 | 1 |
| vomiting | Mean | 1 | 0 | 0 | 0 | 1 | 0 | 0 | 0 |
|  | Standard Deviation | 2 | 1 | 0 | 1 | 2 | 1 | 0 | 0 |
| fever | Mean | 4 | 2 | 1 | 1 | 5 | 2 | 1 | 0 |
|  | Standard Deviation | 4 | 3 | 2 | 1 | 4 | 3 | 2 | 1 |
| chills and shiver | Mean | 4 | 2 | 1 | 0 | 5 | 2 | 1 | 0 |
|  | Standard Deviation | 4 | 3 | 3 | 1 | 4 | 3 | 2 | 1 |
| local pain in hand | Mean | 5 | 3 | 5 | 3 | 5 | 4 | 5 | 2 |
|  | Standard Deviation | 3 | 3 | 3 | 3 | 3 | 3 | 4 | 3 |
| pain in foot | Mean | 3 | 2 | 2 | 1 | 3 | 2 | 0 | 2 |
|  | Standard Deviation | 4 | 3 | 4 | 2 | 4 | 3 | 0 | 3 |
| cellulitis warmth and swollen armpit glands | Mean | 0 | 0 | 0 | 0 | 1 | 0 | 0 | 0 |
|  | Standard Deviation | 1 | 1 | 0 | 0 | 2 | 1 | 0 | 0 |
| loss of appetite | Mean | 2 | 1 | 1 | 1 | 3 | 1 | 0 | 0 |
|  | Standard Deviation | 3 | 2 | 2 | 1 | 3 | 2 | 1 | 1 |
| dizziness | Mean | 2 | 1 | 1 | 1 | 2 | 1 | 1 | 1 |
|  | Standard Deviation | 3 | 2 | 1 | 1 | 3 | 2 | 1 | 2 |
| redness and itch | Mean | 0 | 0 | 0 | 0 | 1 | 0 | 0 | 0 |
|  | Standard Deviation | 1 | 1 | 0 | 1 | 2 | 1 | 0 | 0 |
| Crump/ abdominal pain | Mean | 1 | 1 | 1 | 0 | 1 | 0 | 1 | 0 |
|  | Standard Deviation | 2 | 2 | 2 | 1 | 2 | 1 | 2 | 1 |
| arthralgia | Mean | 3 | 2 | 2 | 1 | 3 | 2 | 1 | 0 |
|  | Standard Deviation | 4 | 3 | 3 | 2 | 4 | 3 | 2 | 1 |
| chest pain | Mean | 1 | 1 | 0 | 0 | 1 | 1 | 0 | 0 |
|  | Standard Deviation | 2 | 2 | 1 | 1 | 2 | 2 | 1 | 0 |
| Cough | Mean | 1 | 0 | 0 | 0 | 1 | 0 | 0 | 0 |
|  | Standard Deviation | 2 | 1 | 0 | 1 | 2 | 2 | 0 | 1 |
| shortness of breath | Mean | 1 | 0 | 0 | 0 | 1 | 0 | 0 | 0 |
|  | Standard Deviation | 2 | 1 | 0 | 1 | 2 | 1 | 0 | 0 |
| Blurred vision | Mean | 1 | 0 | 0 | 0 | 1 | 0 | 0 | 0 |
|  | Standard Deviation | 2 | 1 | 0 | 0 | 2 | 1 | 0 | 0 |
| Diplopia | Mean | 0 | 0 | 0 | 0 | 0 | 0 | 0 | 0 |
|  | Standard Deviation | 1 | 1 | 0 | 1 | 1 | 0 | 0 | 0 |
| diarrhea | Mean | 1 | 0 | 1 | 0 | 1 | 0 | 0 | 0 |
|  | Standard Deviation | 2 | 1 | 3 | 1 | 2 | 1 | 0 | 2 |
|  | Standard Deviation | 4 | 3 | 3 | 2 | 3 | 4 | 2 | 2 |
| insomnia | Mean | 2 | 1 | 0 | 0 | 3 | 1 | 0 | 1 |
|  | Standard Deviation | 3 | 2 | 1 | 1 | 3 | 2 | 0 | 1 |
| jaw pain | Mean | 1 | 0 | 0 | 0 | 1 | 0 | 0 | 0 |
|  | Standard Deviation | 2 | 1 | 0 | 1 | 2 | 1 | 0 | 0 |
| Earache | Mean | 1 | 0 | 0 | 0 | 1 | 0 | 0 | 0 |
|  | Standard Deviation | 2 | 1 | 0 | 1 | 2 | 1 | 0 | 0 |
| dysphagia | Mean | 0 | 0 | 0 | 0 | 0 | 0 | 0 | 0 |
|  | Standard Deviation | 1 | 1 | 0 | 1 | 1 | 0 | 0 | 0 |
| facial numbness | Mean | 0 | 0 | 0 | 0 | 0 | 0 | 0 | 0 |
|  | Standard Deviation | 0 | 0 | 0 | 0 | 1 | 0 | 0 | 0 |
| anesthesia (face/body) | Mean | 0 | 0 | 0 | 0 | 0 | 0 | 0 | 0 |
|  | Standard Deviation | 1 | 1 | 1 | 1 | 2 | 1 | 0 | 0 |
| Bradycardia | Mean | 2 | 1 | 0 | 0 | 2 | 1 | 1 | 1 |
|  | Standard Deviation | 3 | 2 | 1 | 1 | 3 | 2 | 1 | 2 |
| Tachycardia | Mean | 0 | 0 | 0 | 0 | 0 | 0 | 0 | 0 |
|  | Standard Deviation | 1 | 0 | 0 | 1 | 1 | 0 | 0 | 0 |
| Anaphylactic shock | Mean | 0 | 0 | 0 | 0 | 0 | 0 | 0 | 0 |
|  | Standard Deviation | 0 | 0 | 0 | 0 | 1 | 0 | 0 | 0 |
| thrombosis and blood-clotting | Mean | 0 | 0 | 0 | 0 | 0 | 0 | 0 | 0 |
|  | Standard Deviation | 0 | 0 | 0 | 0 | 1 | 0 | 0 | 0 |
| Oral bleeding | Mean | 0 | 0 | 0 | 0 | 0 | 0 | 0 | 0 |
|  | Standard Deviation | 0 | 0 | 0 | 0 | 0 | 0 | 0 | 0 |
| Nasal bleeding | Mean | 0 | 0 | 0 | 0 | 0 | 0 | 0 | 0 |
|  | Standard Deviation | 0 | 1 | 0 | 0 | 1 | 1 | 0 | 0 |
| Seizure | Mean | 0 | 0 | 0 | 0 | 0 | 0 | 0 | 0 |
|  | Standard Deviation | 1 | 0 | 0 | 0 | 0 | 0 | 0 | 0 |
| Faint | Mean | 0 | 0 | 0 | 0 | 0 | 0 | 0 | 0 |
|  | Standard Deviation | 1 | 1 | 0 | 0 | 1 | 1 | 0 | 0 |
| Optic neuritis | Mean | 0 | 0 | 0 | 0 | 0 | 0 | 0 | 0 |
|  | Standard Deviation | 1 | 0 | 0 | 0 | 0 | 0 | 0 | 0 |
| Speech disorders | Mean | 0 | 0 | 0 | 0 | 0 | 0 | 0 | 0 |
|  | Standard Deviation | 0 | 1 | 0 | 0 | 1 | 0 | 0 | 0 |

Table. The mean and standard deviation of symptoms in patients with/without a history of infection (Influenza, SARS, MERS, and EBV) (based on the type of vaccine used.

|  | | Vaccine | | | | Vaccine | | | | Vaccine | | | | Vaccine | | | |
| --- | --- | --- | --- | --- | --- | --- | --- | --- | --- | --- | --- | --- | --- | --- | --- | --- | --- |
|  |  | Astrazeneca | Sputnic V | Covaxine | Sinopharm | Astrazeneca | Sputnic V | Covaxine | Sinopharm | Astrazeneca | Sputnic V | Covaxine | Sinopharm | Astrazeneca | Sputnic V | Covaxine | Sinopharm |
| Fatigue | Mean | 6.10 | 3.51 | 2.67 | 1.83 | 8.00 | . | . | . | 4.91 | 4.25 | . | 3.00 | 5.50 | 3.23 | 3.16 | 2.14 |
|  | Standard Deviation | 2.95 | 3.25 | 2.42 | 2.66 | . | . | . | . | 3.75 | 4.43 | . | 2.83 | 3.10 | 3.18 | 3.32 | 2.59 |
|  | Minimum | .00 | .00 | 1.00 | .00 | 8.00 | . | . | . | .00 | .00 | . | 1.00 | .00 | .00 | .00 | .00 |
|  | Maximum | 10.00 | 10.00 | 7.00 | 9.00 | 8.00 | . | . | . | 10.00 | 9.00 | . | 5.00 | 10.00 | 10.00 | 8.00 | 8.00 |
| Malaise | Mean | 5.80 | 3.07 | 1.67 | 1.38 | 7.00 | . | . | . | 3.36 | 4.50 | . | 1.00 | 5.08 | 2.69 | 2.68 | 1.46 |
|  | Standard Deviation | 3.18 | 3.17 | 1.75 | 2.69 | . | . | . | . | 3.11 | 4.65 | . | .00 | 3.21 | 2.91 | 3.00 | 2.07 |
|  | Minimum | .00 | .00 | .00 | .00 | 7.00 | . | . | . | .00 | .00 | . | 1.00 | .00 | .00 | .00 | .00 |
|  | Maximum | 10.00 | 10.00 | 5.00 | 9.00 | 7.00 | . | . | . | 7.00 | 9.00 | . | 1.00 | 10.00 | 10.00 | 9.00 | 7.00 |
| Headache | Mean | 4.81 | 2.76 | 1.17 | 1.76 | 7.00 | . | . | . | 2.45 | 2.50 | . | .00 | 4.36 | 1.99 | 2.26 | 1.51 |
|  | Standard Deviation | 3.66 | 3.31 | 2.40 | 2.97 | . | . | . | . | 3.01 | 2.38 | . | .00 | 3.61 | 3.00 | 2.83 | 2.63 |
|  | Minimum | .00 | .00 | .00 | .00 | 7.00 | . | . | . | .00 | 1.00 | . | .00 | .00 | .00 | .00 | .00 |
|  | Maximum | 10.00 | 10.00 | 6.00 | 10.00 | 7.00 | . | . | . | 8.00 | 6.00 | . | .00 | 10.00 | 10.00 | 9.00 | 10.00 |
| Body pain | Mean | 5.78 | 3.26 | 3.00 | 1.28 | 10.00 | . | . | . | 5.18 | 1.50 | . | .50 | 5.62 | 3.02 | 2.42 | 1.30 |
|  | Standard Deviation | 3.57 | 3.53 | 3.16 | 2.17 | . | . | . | . | 3.97 | 1.91 | . | .71 | 3.51 | 3.42 | 3.53 | 2.26 |
|  | Minimum | .00 | .00 | .00 | .00 | 10.00 | . | . | . | .00 | .00 | . | .00 | .00 | .00 | .00 | .00 |
|  | Maximum | 10.00 | 10.00 | 9.00 | 7.00 | 10.00 | . | . | . | 10.00 | 4.00 | . | 1.00 | 10.00 | 10.00 | 10.00 | 10.00 |
| Fever | Mean | 5.00 | 1.94 | 1.00 | .45 | 7.00 | . | . | . | 4.45 | 1.75 | . | .00 | 4.40 | 1.76 | .89 | .56 |
|  | Standard Deviation | 3.62 | 3.00 | 1.67 | 1.21 | . | . | . | . | 4.30 | 2.87 | . | .00 | 3.66 | 2.74 | 2.38 | 1.05 |
|  | Minimum | .00 | .00 | .00 | .00 | 7.00 | . | . | . | .00 | .00 | . | .00 | .00 | .00 | .00 | .00 |
|  | Maximum | 10.00 | 10.00 | 4.00 | 6.00 | 7.00 | . | . | . | 10.00 | 6.00 | . | .00 | 10.00 | 10.00 | 10.00 | 5.00 |
| Chills and shiver | Mean | 4.78 | 2.06 | .83 | .41 | 2.00 | . | . | . | 3.64 | 1.50 | . | .50 | 4.05 | 1.67 | 1.05 | .46 |
|  | Standard Deviation | 3.92 | 3.35 | 2.04 | 1.32 | . | . | . | . | 3.61 | 3.00 | . | .71 | 3.80 | 2.95 | 2.76 | 1.37 |
|  | Minimum | .00 | .00 | .00 | .00 | 2.00 | . | . | . | .00 | .00 | . | .00 | .00 | .00 | .00 | .00 |
|  | Maximum | 10.00 | 10.00 | 5.00 | 6.00 | 2.00 | . | . | . | 10.00 | 6.00 | . | 1.00 | 10.00 | 10.00 | 10.00 | 7.00 |
| Local pain in hand | Mean | 5.49 | 3.51 | 5.00 | 2.38 | 9.00 | . | . | . | 2.09 | 4.50 | . | 3.00 | 4.89 | 3.22 | 4.74 | 2.72 |
|  | Standard Deviation | 3.37 | 3.40 | 3.58 | 2.58 | . | . | . | . | 2.55 | 3.11 | . | 4.24 | 3.38 | 3.38 | 2.96 | 3.04 |
|  | Minimum | .00 | .00 | .00 | .00 | 9.00 | . | . | . | .00 | 1.00 | . | .00 | .00 | .00 | .00 | .00 |
|  | Maximum | 10.00 | 10.00 | 10.00 | 10.00 | 9.00 | . | . | . | 8.00 | 8.00 | . | 6.00 | 10.00 | 10.00 | 10.00 | 10.00 |
| Pain in foot | Mean | 3.43 | 1.84 | .00 | 1.83 | 9.00 | . | . | . | 2.09 | .50 | . | .00 | 3.09 | 1.59 | 2.42 | .62 |
|  | Standard Deviation | 3.77 | 3.03 | .00 | 3.00 | . | . | . | . | 3.24 | .58 | . | .00 | 3.61 | 2.93 | 3.70 | 1.59 |
|  | Minimum | .00 | .00 | .00 | .00 | 9.00 | . | . | . | .00 | .00 | . | .00 | .00 | .00 | .00 | .00 |
|  | Maximum | 10.00 | 10.00 | .00 | 10.00 | 9.00 | . | . | . | 10.00 | 1.00 | . | .00 | 10.00 | 10.00 | 10.00 | 7.00 |
| Loss of appetite | Mean | 2.71 | .75 | .33 | .34 | 5.00 | . | . | . | 1.82 | 1.00 | . | .00 | 2.23 | .81 | 1.21 | .56 |
|  | Standard Deviation | 3.25 | 1.84 | .52 | .90 | . | . | . | . | 2.48 | 2.00 | . | .00 | 2.98 | 1.80 | 2.30 | 1.40 |
|  | Minimum | .00 | .00 | .00 | .00 | 5.00 | . | . | . | .00 | .00 | . | .00 | .00 | .00 | .00 | .00 |
|  | Maximum | 10.00 | 8.00 | 1.00 | 4.00 | 5.00 | . | . | . | 7.00 | 4.00 | . | .00 | 10.00 | 10.00 | 8.00 | 6.00 |
| Dizziness | Mean | 2.52 | 1.14 | .83 | .83 | .00 | . | . | . | 1.91 | 4.50 | . | 1.00 | 2.31 | .97 | .63 | .55 |
|  | Standard Deviation | 3.14 | 2.28 | 1.33 | 1.83 | . | . | . | . | 3.18 | 5.26 | . | 1.41 | 3.09 | 1.99 | 1.42 | 1.23 |
|  | Minimum | .00 | .00 | .00 | .00 | .00 | . | . | . | .00 | .00 | . | .00 | .00 | .00 | .00 | .00 |
|  | Maximum | 10.00 | 10.00 | 3.00 | 7.00 | .00 | . | . | . | 10.00 | 10.00 | . | 2.00 | 10.00 | 10.00 | 5.00 | 5.00 |
| Arthralgia | Mean | 3.17 | 2.20 | 1.17 | .38 | 9.00 | . | . | . | 2.91 | .75 | . | .00 | 3.08 | 1.72 | 1.68 | .68 |
|  | Standard Deviation | 3.56 | 3.07 | 1.60 | .78 | . | . | . | . | 3.81 | 1.50 | . | .00 | 3.51 | 2.91 | 2.89 | 1.70 |
|  | Minimum | .00 | .00 | .00 | .00 | 9.00 | . | . | . | .00 | .00 | . | .00 | .00 | .00 | .00 | .00 |
|  | Maximum | 10.00 | 10.00 | 4.00 | 3.00 | 9.00 | . | . | . | 10.00 | 3.00 | . | .00 | 10.00 | 10.00 | 10.00 | 8.00 |
| Insomnia | Mean | 2.56 | 1.18 | .00 | .48 | 8.00 | . | . | . | 2.91 | .00 | . | 1.50 | 2.20 | 1.25 | .37 | .45 |
|  | Standard Deviation | 3.27 | 2.47 | .00 | 1.21 | . | . | . | . | 3.86 | .00 | . | 2.12 | 3.11 | 2.47 | 1.38 | 1.23 |
|  | Minimum | .00 | .00 | .00 | .00 | 8.00 | . | . | . | .00 | .00 | . | .00 | .00 | .00 | .00 | .00 |
|  | Maximum | 10.00 | 10.00 | .00 | 5.00 | 8.00 | . | . | . | 10.00 | .00 | . | 3.00 | 10.00 | 10.00 | 6.00 | 5.00 |
| Bradycardia | Mean | 1.60 | 1.06 | .50 | .72 | 7.00 | . | . | . | .55 | 2.75 | . | .00 | 1.59 | .76 | .21 | .34 |
|  | Standard Deviation | 2.59 | 2.35 | .84 | 1.94 | . | . | . | . | 1.81 | 3.20 | . | .00 | 2.74 | 1.93 | .71 | .98 |
|  | Minimum | .00 | .00 | .00 | .00 | 7.00 | . | . | . | .00 | .00 | . | .00 | .00 | .00 | .00 | .00 |
|  | Maximum | 10.00 | 10.00 | 2.00 | 10.00 | 7.00 | . | . | . | 6.00 | 6.00 | . | .00 | 10.00 | 10.00 | 3.00 | 5.00 |
| Crump/abdominal pain | Mean | 1.25 | .39 | .83 | .21 | 3.00 | . | . | . | 1.09 | 2.50 | . | .00 | 1.00 | .51 | .89 | .30 |
|  | Standard Deviation | 2.38 | 1.31 | 2.04 | .68 | . | . | . | . | 2.98 | 3.32 | . | .00 | 2.13 | 1.52 | 2.16 | 1.10 |
|  | Minimum | .00 | .00 | .00 | .00 | 3.00 | . | . | . | .00 | .00 | . | .00 | .00 | .00 | .00 | .00 |
|  | Maximum | 10.00 | 8.00 | 5.00 | 3.00 | 3.00 | . | . | . | 10.00 | 7.00 | . | .00 | 10.00 | 10.00 | 8.00 | 7.00 |
| Chest pain | Mean | 1.42 | .83 | .33 | .14 | 6.00 | . | . | . | 1.00 | .00 | . | .00 | 1.20 | .62 | .47 | .24 |
|  | Standard Deviation | 2.43 | 2.02 | .82 | .44 | . | . | . | . | 2.10 | .00 | . | .00 | 2.49 | 1.78 | 1.31 | .92 |
|  | Minimum | .00 | .00 | .00 | .00 | 6.00 | . | . | . | .00 | .00 | . | .00 | .00 | .00 | .00 | .00 |
|  | Maximum | 10.00 | 10.00 | 2.00 | 2.00 | 6.00 | . | . | . | 7.00 | .00 | . | .00 | 10.00 | 10.00 | 5.00 | 5.00 |
| Diarrhea | Mean | .78 | .33 | .00 | .38 | 1.00 | . | . | . | .73 | .00 | . | .00 | .72 | .33 | .89 | .10 |
|  | Standard Deviation | 1.96 | 1.10 | .00 | 1.86 | . | . | . | . | 2.41 | .00 | . | .00 | 1.97 | 1.26 | 2.73 | .61 |
|  | Minimum | .00 | .00 | .00 | .00 | 1.00 | . | . | . | .00 | .00 | . | .00 | .00 | .00 | .00 | .00 |
|  | Maximum | 10.00 | 8.00 | .00 | 10.00 | 1.00 | . | . | . | 8.00 | .00 | . | .00 | 10.00 | 10.00 | 10.00 | 5.00 |
| Cough | Mean | .99 | .50 | .00 | .31 | 7.00 | . | . | . | 1.09 | .00 | . | .00 | .71 | .32 | .00 | .21 |
|  | Standard Deviation | 2.11 | 1.53 | .00 | 1.00 | . | . | . | . | 2.98 | .00 | . | .00 | 1.71 | 1.11 | .00 | .65 |
|  | Minimum | .00 | .00 | .00 | .00 | 7.00 | . | . | . | .00 | .00 | . | .00 | .00 | .00 | .00 | .00 |
|  | Maximum | 10.00 | 10.00 | .00 | 5.00 | 7.00 | . | . | . | 10.00 | .00 | . | .00 | 10.00 | 8.00 | .00 | 4.00 |
| Shortness of breath | Mean | .94 | .44 | .17 | .03 | 7.00 | . | . | . | 1.36 | .00 | . | .00 | .71 | .36 | .00 | .23 |
|  | Standard Deviation | 1.88 | 1.36 | .41 | .19 | . | . | . | . | 3.23 | .00 | . | .00 | 1.75 | 1.33 | .00 | 1.17 |
|  | Minimum | .00 | .00 | .00 | .00 | 7.00 | . | . | . | .00 | .00 | . | .00 | .00 | .00 | .00 | .00 |
|  | Maximum | 10.00 | 10.00 | 1.00 | 1.00 | 7.00 | . | . | . | 10.00 | .00 | . | .00 | 10.00 | 10.00 | .00 | 9.00 |
| Vision disorders | Mean | .90 | .32 | .17 | .28 | .00 | . | . | . | 1.00 | .00 | . | .00 | .74 | .41 | .05 | .21 |
|  | Standard Deviation | 1.89 | 1.20 | .41 | .92 | . | . | . | . | 1.79 | .00 | . | .00 | 1.81 | 1.42 | .23 | .97 |
|  | Minimum | .00 | .00 | .00 | .00 | .00 | . | . | . | .00 | .00 | . | .00 | .00 | .00 | .00 | .00 |
|  | Maximum | 8.00 | 9.00 | 1.00 | 4.00 | .00 | . | . | . | 5.00 | .00 | . | .00 | 10.00 | 9.00 | 1.00 | 7.00 |
| Jaw pain | Mean | .84 | .30 | .00 | .00 | 7.00 | . | . | . | 1.45 | 1.25 | . | .00 | .60 | .26 | .05 | .34 |
|  | Standard Deviation | 2.11 | 1.21 | .00 | .00 | . | . | . | . | 3.36 | 2.50 | . | .00 | 1.81 | 1.03 | .23 | 1.36 |
|  | Minimum | .00 | .00 | .00 | .00 | 7.00 | . | . | . | .00 | .00 | . | .00 | .00 | .00 | .00 | .00 |
|  | Maximum | 10.00 | 10.00 | .00 | .00 | 7.00 | . | . | . | 10.00 | 5.00 | . | .00 | 10.00 | 7.00 | 1.00 | 9.00 |
| Earache | Mean | .75 | .21 | .00 | .07 | 7.00 | . | . | . | .55 | 1.75 | . | .00 | .56 | .33 | .00 | .23 |
|  | Standard Deviation | 2.00 | .86 | .00 | .37 | . | . | . | . | 1.04 | 3.50 | . | .00 | 1.81 | 1.31 | .00 | 1.04 |
|  | Minimum | .00 | .00 | .00 | .00 | 7.00 | . | . | . | .00 | .00 | . | .00 | .00 | .00 | .00 | .00 |
|  | Maximum | 10.00 | 7.00 | .00 | 2.00 | 7.00 | . | . | . | 3.00 | 7.00 | . | .00 | 10.00 | 9.00 | .00 | 8.00 |
| Vomiting | Mean | .61 | .36 | .17 | .07 | .00 | . | . | . | .00 | .00 | . | .00 | .55 | .21 | .00 | .28 |
|  | Standard Deviation | 1.80 | 1.36 | .41 | .37 | . | . | . | . | .00 | .00 | . | .00 | 1.91 | 1.09 | .00 | 1.33 |
|  | Minimum | .00 | .00 | .00 | .00 | .00 | . | . | . | .00 | .00 | . | .00 | .00 | .00 | .00 | .00 |
|  | Maximum | 10.00 | 8.00 | 1.00 | 2.00 | .00 | . | . | . | .00 | .00 | . | .00 | 10.00 | 10.00 | .00 | 10.00 |
| Cellulitis warmth and swollen armpit glands | Mean | .56 | .30 | .17 | .10 | 1.00 | . | . | . | .91 | .00 | . | .00 | .20 | .12 | .00 | .06 |
|  | Standard Deviation | 1.56 | 1.02 | .41 | .31 | . | . | . | . | 3.02 | .00 | . | .00 | .90 | .60 | .00 | .23 |
|  | Minimum | .00 | .00 | .00 | .00 | 1.00 | . | . | . | .00 | .00 | . | .00 | .00 | .00 | .00 | .00 |
|  | Maximum | 8.00 | 5.00 | 1.00 | 1.00 | 1.00 | . | . | . | 10.00 | .00 | . | .00 | 10.00 | 6.00 | .00 | 1.00 |
| Redness and itch | Mean | .52 | .43 | .17 | .00 | .00 | . | . | . | .91 | .50 | . | .00 | .42 | .27 | .11 | .17 |
|  | Standard Deviation | 1.52 | 1.34 | .41 | .00 | . | . | . | . | 1.81 | 1.00 | . | .00 | 1.35 | 1.15 | .32 | .88 |
|  | Minimum | .00 | .00 | .00 | .00 | .00 | . | . | . | .00 | .00 | . | .00 | .00 | .00 | .00 | .00 |
|  | Maximum | 10.00 | 8.00 | 1.00 | .00 | .00 | . | . | . | 5.00 | 2.00 | . | .00 | 10.00 | 10.00 | 1.00 | 7.00 |
| Blurred vision | Mean | .71 | .22 | .00 | .00 | .00 | . | . | . | 1.36 | .00 | . | .00 | .56 | .27 | .00 | .08 |
|  | Standard Deviation | 1.79 | .84 | .00 | .00 | . | . | . | . | 3.23 | .00 | . | .00 | 1.63 | 1.16 | .00 | .50 |
|  | Minimum | .00 | .00 | .00 | .00 | .00 | . | . | . | .00 | .00 | . | .00 | .00 | .00 | .00 | .00 |
|  | Maximum | 10.00 | 6.00 | .00 | .00 | .00 | . | . | . | 10.00 | .00 | . | .00 | 10.00 | 8.00 | .00 | 4.00 |
| Diplopia | Mean | .27 | .08 | .00 | .00 | .00 | . | . | . | .91 | .00 | . | .00 | .22 | .14 | .00 | .10 |
|  | Standard Deviation | 1.25 | .43 | .00 | .00 | . | . | . | . | 1.81 | .00 | . | .00 | 1.02 | .79 | .00 | .51 |
|  | Minimum | .00 | .00 | .00 | .00 | .00 | . | . | . | .00 | .00 | . | .00 | .00 | .00 | .00 | .00 |
|  | Maximum | 10.00 | 3.00 | .00 | .00 | .00 | . | . | . | 5.00 | .00 | . | .00 | 10.00 | 6.00 | .00 | 4.00 |
| Dysphagia | Mean | .28 | .12 | .00 | .00 | .00 | . | . | . | .36 | .00 | . | .00 | .30 | .09 | .00 | .27 |
|  | Standard Deviation | 1.08 | .45 | .00 | .00 | . | . | . | . | 1.21 | .00 | . | .00 | 1.33 | .53 | .00 | 1.25 |
|  | Minimum | .00 | .00 | .00 | .00 | .00 | . | . | . | .00 | .00 | . | .00 | .00 | .00 | .00 | .00 |
|  | Maximum | 7.00 | 3.00 | .00 | .00 | .00 | . | . | . | 4.00 | .00 | . | .00 | 10.00 | 5.00 | .00 | 9.00 |
| Facial numbness | Mean | .07 | .06 | .00 | .00 | .00 | . | . | . | .09 | .00 | . | .00 | .02 | .02 | .00 | .00 |
|  | Standard Deviation | .73 | .33 | .00 | .00 | . | . | . | . | .30 | .00 | . | .00 | .18 | .13 | .00 | .00 |
|  | Minimum | .00 | .00 | .00 | .00 | .00 | . | . | . | .00 | .00 | . | .00 | .00 | .00 | .00 | .00 |
|  | Maximum | 9.00 | 3.00 | .00 | .00 | .00 | . | . | . | 1.00 | .00 | . | .00 | 3.00 | 1.00 | .00 | .00 |
| Anesthesia (face/body) | Mean | .38 | .12 | .00 | .03 | 8.00 | . | . | . | .00 | .00 | . | .50 | .22 | .19 | .26 | .18 |
|  | Standard Deviation | 1.47 | .71 | .00 | .19 | . | . | . | . | .00 | .00 | . | .71 | 1.04 | 1.01 | .93 | .70 |
|  | Minimum | .00 | .00 | .00 | .00 | 8.00 | . | . | . | .00 | .00 | . | .00 | .00 | .00 | .00 | .00 |
|  | Maximum | 10.00 | 7.00 | .00 | 1.00 | 8.00 | . | . | . | .00 | .00 | . | 1.00 | 10.00 | 9.00 | 4.00 | 4.00 |
| Tachycardia | Mean | .13 | .05 | .17 | .00 | .00 | . | . | . | .00 | .00 | . | .00 | .15 | .05 | .00 | .10 |
|  | Standard Deviation | .57 | .21 | .41 | .00 | . | . | . | . | .00 | .00 | . | .00 | .93 | .37 | .00 | .83 |
|  | Minimum | .00 | .00 | .00 | .00 | .00 | . | . | . | .00 | .00 | . | .00 | .00 | .00 | .00 | .00 |
|  | Maximum | 5.00 | 1.00 | 1.00 | .00 | .00 | . | . | . | .00 | .00 | . | .00 | 10.00 | 5.00 | .00 | 7.00 |
| Anaphylactic shock | Mean | .07 | .02 | .00 | .00 | .00 | . | . | . | .00 | .00 | . | .00 | .01 | .01 | .00 | .00 |
|  | Standard Deviation | .54 | .13 | .00 | .00 | . | . | . | . | .00 | .00 | . | .00 | .09 | .13 | .00 | .00 |
|  | Minimum | .00 | .00 | .00 | .00 | .00 | . | . | . | .00 | .00 | . | .00 | .00 | .00 | .00 | .00 |
|  | Maximum | 6.00 | 1.00 | .00 | .00 | .00 | . | . | . | .00 | .00 | . | .00 | 1.00 | 2.00 | .00 | .00 |
| Thrombosis and blood-clotting | Mean | .13 | .02 | .00 | .00 | .00 | . | . | . | .00 | .00 | . | .00 | .02 | .01 | .00 | .04 |
|  | Standard Deviation | .92 | .15 | .00 | .00 | . | . | . | . | .00 | .00 | . | .00 | .13 | .12 | .00 | .20 |
|  | Minimum | .00 | .00 | .00 | .00 | .00 | . | . | . | .00 | .00 | . | .00 | .00 | .00 | .00 | .00 |
|  | Maximum | 10.00 | 1.00 | .00 | .00 | .00 | . | . | . | .00 | .00 | . | .00 | 1.00 | 1.00 | .00 | 1.00 |
| Oral bleeding | Mean | .05 | .01 | .00 | .00 | .00 | . | . | . | .00 | .00 | . | .00 | .02 | .01 | .05 | .01 |
|  | Standard Deviation | .35 | .09 | .00 | .00 | . | . | . | . | .00 | .00 | . | .00 | .27 | .10 | .23 | .12 |
|  | Minimum | .00 | .00 | .00 | .00 | .00 | . | . | . | .00 | .00 | . | .00 | .00 | .00 | .00 | .00 |
|  | Maximum | 4.00 | 1.00 | .00 | .00 | .00 | . | . | . | .00 | .00 | . | .00 | 5.00 | 1.00 | 1.00 | 1.00 |
| Nasal bleeding | Mean | .11 | .10 | .00 | .00 | .00 | . | . | . | .73 | .00 | . | .00 | .06 | .06 | .05 | .01 |
|  | Standard Deviation | .64 | .56 | .00 | .00 | . | . | . | . | 2.10 | .00 | . | .00 | .46 | .52 | .23 | .12 |
|  | Minimum | .00 | .00 | .00 | .00 | .00 | . | . | . | .00 | .00 | . | .00 | .00 | .00 | .00 | .00 |
|  | Maximum | 5.00 | 5.00 | .00 | .00 | .00 | . | . | . | 7.00 | .00 | . | .00 | 8.00 | 8.00 | 1.00 | 1.00 |
| Seizure | Mean | .01 | .02 | .00 | .00 | .00 | . | . | . | .00 | .00 | . | .00 | .04 | .01 | .00 | .00 |
|  | Standard Deviation | .08 | .18 | .00 | .00 | . | . | . | . | .00 | .00 | . | .00 | .51 | .10 | .00 | .00 |
|  | Minimum | .00 | .00 | .00 | .00 | .00 | . | . | . | .00 | .00 | . | .00 | .00 | .00 | .00 | .00 |
|  | Maximum | 1.00 | 2.00 | .00 | .00 | .00 | . | . | . | .00 | .00 | . | .00 | 10.00 | 1.00 | .00 | .00 |
| Faint | Mean | .24 | .13 | .00 | .00 | .00 | . | . | . | .91 | .00 | . | .00 | .10 | .07 | .00 | .00 |
|  | Standard Deviation | 1.03 | .65 | .00 | .00 | . | . | . | . | 3.02 | .00 | . | .00 | .81 | .68 | .00 | .00 |
|  | Minimum | .00 | .00 | .00 | .00 | .00 | . | . | . | .00 | .00 | . | .00 | .00 | .00 | .00 | .00 |
|  | Maximum | 6.00 | 5.00 | .00 | .00 | .00 | . | . | . | 10.00 | .00 | . | .00 | 10.00 | 8.00 | .00 | .00 |
| Optic neuritis | Mean | .05 | .04 | .00 | .00 | .00 | . | . | . | .00 | .00 | . | .00 | .08 | .02 | .00 | .00 |
|  | Standard Deviation | .27 | .23 | .00 | .00 | . | . | . | . | .00 | .00 | . | .00 | .62 | .16 | .00 | .00 |
|  | Minimum | .00 | .00 | .00 | .00 | .00 | . | . | . | .00 | .00 | . | .00 | .00 | .00 | .00 | .00 |
|  | Maximum | 2.00 | 2.00 | .00 | .00 | .00 | . | . | . | .00 | .00 | . | .00 | 9.00 | 2.00 | .00 | .00 |
| Speech disorders | Mean | .13 | .04 | .00 | .00 | .00 | . | . | . | .00 | .00 | . | .00 | .07 | .06 | .00 | .01 |
|  | Standard Deviation | .78 | .20 | .00 | .00 | . | . | . | . | .00 | .00 | . | .00 | .41 | .66 | .00 | .12 |
|  | Minimum | .00 | .00 | .00 | .00 | .00 | . | . | . | .00 | .00 | . | .00 | .00 | .00 | .00 | .00 |
|  | Maximum | 8.00 | 1.00 | .00 | .00 | .00 | . | . | . | .00 | .00 | . | .00 | 6.00 | 10.00 | .00 | 1.00 |

Table. The mean and standard deviation of symptoms in patients with/without a history of infection (Influenza, SARS, MERS, and EBV) based on the type of vaccine used.

Total

|  | **P-value** | **OR** | **95% CI** | |
| --- | --- | --- | --- | --- |
|  |  |  | **L** | **U** |
| Chills and shiver(1) | .059 | 1.341 | .989 | 1.817 |
| Loss of appetite(1) | .079 | 1.318 | .969 | 1.793 |
| Cellulitis warmth and swollen armpit glands(1) | .063 | 1.540 | .976 | 2.430 |
| Facial numbness(1) | .066 | .150 | .020 | 1.131 |

AstraZeneca

|  | **P-value** | | **OR** | **95% CI** | |
| --- | --- | --- | --- | --- | --- |
|  |  |  |  | **L** | **U** |
| Fatigue(1) | | .013 | .438 | .229 | .837 |
| Loss of appetite(1) | | .022 | 1.642 | 1.073 | 2.514 |
| Chest pain(1) | | .020 | 1.753 | 1.092 | 2.812 |
| Shortness of breath(1) | | .032 | .562 | .331 | .952 |
| Cellulitis warmth and swollen armpit glands(1) | | .071 | 1.711 | .955 | 3.066 |

Sputnik

|  | **P-value** | **OR** | **95% CI** | | |
| --- | --- | --- | --- | --- | --- |
|  |  |  | **L** | **U** | |
| Local pain in hand(1) | .088 | 1.648 | .929 | | 2.925 |
| Shortness of breath(1) | .092 | 1.935 | .897 | | 4.176 |
| Chills and shiver(1) | .006 | 2.093 | 1.232 | | 3.555 |
| Insomnia(1) | .072 | 1.674 | .954 | | 2.936 |
| Vomiting(1) | .064 | .337 | .107 | | 1.065 |
| Dysphagia(1) | .031 | .200 | .046 | | .862 |
| Tachycardia(1) | .064 | .189 | .032 | | 1.101 |
| Faint(1) | .027 | 4.527 | 1.190 | | 17.221 |

Sinopharm

|  | **P-value** | **OR** | **95% CI** | |  |
| --- | --- | --- | --- | --- | --- |
|  |  |  | **L** | **U** |  |
| Body pain(1) | .018 | 3.402 | 1.235 | 9.371 | |
| Headache(1) | .046 | .344 | .121 | .980 | |

Table. Logistic regression model using backward method (history of covid infection)
